# Localized Theta-Burst Magnetic Stimulation Induces Bidirectional Neural Modulation in the Mouse Auditory Cortex In Vivo

**DOI:** 10.1523/ENEURO.0577-24.2025

**Published:** 2025-05-02

**Authors:** Takahiro Yoshikawa, Takashi Tateno

**Affiliations:** ^1^Bioengineering and Bioinformatics, Graduate School of Information Science and Technology, Hokkaido University, Sapporo 060-0814, Japan; ^2^Division of Bioengineering and Bioinformatics, Faculty of Information Science and Technology, Hokkaido University, Sapporo 060-0814, Japan

**Keywords:** auditory cortex, electrophysiology, modulation, rodent, theta-burst magnetic stimulation, transcranial magnetic stimulation

## Abstract

Repetitive transcranial magnetic stimulation (rTMS) is a noninvasive method that has been used to treat various brain disorders. The modulatory effects of rTMS can be adjusted by changing the repetition patterns. Theta-burst magnetic stimulation (TBS) is a magnetic stimulation pattern that can induce long-lasting modulatory effects with a short stimulation period. However, its effects on auditory brain regions remain unclear because of a lack of animal studies in which invasive techniques allow for a detailed exploration of the underlying neural mechanisms. In the current study, we investigated the effects of TBS on the C57BL/6J mouse auditory cortex using a custom-built 7 mm magnetic stimulation coil. Extracellular recordings were made before, during, and after the application of intermittent TBS (iTBS), continuous TBS (cTBS), or sham stimulation. Local field potential amplitudes were increased for 5–20 min post-iTBS compared with the sham condition and were decreased at 10 min post-cTBS compared with the sham condition. The bidirectional modulatory effects observed in our study are consistent with previous findings from other brain regions. Additionally, multiunit activities were significantly altered in cortical layers 2/3 and 4 but not layer 5, indicating that the modulatory effects were localized to the surface region of the auditory cortex. Interestingly, in the iTBS group, the amplitude of average spike waveforms increased with a 15 min delay. Our findings provide physiological evidence of TBS modulation of the rodent auditory cortex and may guide future research seeking to optimize rTMS for modulating hearing abilities.

## Significance Statement

Theta-burst magnetic stimulation (TBS) shows promise for inducing long-lasting neural modulation with short stimulation duration. However, the majority of findings come from studies targeting the motor cortex, and our understanding of its effects in sensory cortical regions remains relatively limited. Additionally, many rodent studies have used human-specific coils that stimulate the entire rodent brain, thus complicating the identification of brain regions responsible for modulatory effects. Here, using a specially constructed millimeter-sized coil, we applied localized TBS to the mouse auditory cortex and successfully induced a bidirectional modulatory effect. Given the limited number of studies assessing the effects of TBS in the mouse auditory cortex, our findings provide valuable methodological insights as well as physiological evidence of TBS-induced auditory modulation.

## Introduction

Repetitive transcranial magnetic stimulation (rTMS) is a noninvasive brain stimulation technique that uses coils to modulate neural activity. It is approved by the United States Food and Drug Administration for treating disorders such as depression, migraine, obsessive-compulsive disorder, and nicotine addiction (Scheper et al., 2022). After three decades of research, it is now understood that the repetition pattern of rTMS is a key determinant of its modulatory effect (Chervyakov et al., 2015; Bai et al., 2022). rTMS applied at frequencies below 1 Hz is considered low-frequency rTMS, which is known for its inhibitory effects on neural activity (Sebastianelli et al., 2017). In contrast, rTMS with stimulation frequencies above 10 Hz is classified as high-frequency rTMS and has facilitatory effects (Che et al., 2021). Theta-burst magnetic stimulation (TBS), first reported by Huang et al. (2005), is a complex stimulation pattern in which triplet pulses with a 20 ms interpulse interval (50 Hz), called “bursts,” are presented at an interburst interval of 200 ms (5 Hz). Similar to low- and high-frequency rTMS, TBS can be used to induce bidirectional modulatory effects by changing its patterns. Continuous application of TBS is called continuous TBS (cTBS) and produces inhibitory effects (Huang et al., 2005). The addition of 8 s intervals after each 2 s TBS train can change its modulatory effect to a facilitatory effect and is called intermittent TBS (iTBS; Huang et al., 2005). The notable advantage of TBS is its long-lasting effects with a short stimulation duration; only 40 s of cTBS produces 60 min of inhibition, and 200 s of iTBS has facilitatory effects on the brain for 20 min (Huang et al., 2005; Suppa et al., 2016). In clinical settings, however, TBS application is less common than high-frequency rTMS application because it is a relatively new stimulation pattern that requires further evidence and adjustment.

To fully optimize TBS protocols, a bottom-up approach that enhances our understanding of the underlying neuronal mechanisms is crucial. Rodent models offer valuable insights because they allow for invasive experiments. The motor cortex is one of the most common target regions because the resulting modulatory effects can be easily examined using motor-evoked potentials (Klomjai et al., 2015; Jannati et al., 2023). In contrast, studies targeting sensory cortical regions, and especially the auditory cortex, are relatively limited. Several studies have demonstrated the modulatory effects of rTMS in auditory brain regions. For example, Mulders et al. (2016) applied low-intensity rTMS to the auditory cortex of guinea pigs and observed reduced behavioral signs of tinnitus without significantly altering spontaneous firing rates in the inferior colliculus. Later, Mulders et al. (2019) reported that 1 and 10 Hz rTMS to the prefrontal cortex modulates the auditory thalamus, but observed no improvements in tinnitus. It was subsequently reported that facilitatory stimulation patterns (20 Hz and iTBS) applied to the prefrontal cortex can successfully modulate neural activity in the auditory thalamus and reduce tinnitus (Zimdahl et al., 2021). To our knowledge, however, no studies have explored the modulatory effects of TBS on the auditory cortex itself, nor have any used mouse models, which are commonly used and well established in research.

In the present study, we constructed a rodent-specific, millimeter-sized magnetic stimulation (MS) coil and demonstrated modulation of the mouse auditory cortex using low-intensity TBS. Using numerical simulations, we chose an input peak-to-peak voltage that achieves our target electric field strength in layer 4 of the auditory cortex. Electrophysiological activity in the auditory cortex was recorded before, during, and after TBS. Given the limited number of studies reporting neural activity during TBS, we also analyzed peak amplitude changes in MS-evoked potentials (MSEPs) obtained during iTBS and cTBS. To assess the modulatory effects of TBS, neural activity before and after TBS was compared among the iTBS, cTBS, and sham (0 V input to the coil) conditions. Our coil successfully induced a bidirectional modulatory effect in which MSEP peak amplitudes increased for 20 min after iTBS and reduced for 10 min after cTBS. Multiunit activity (MUA) analysis revealed that spike counts decreased for 20 min after cTBS delivery, and spike waveform amplitudes were modulated with a 15 min delay after iTBS delivery. The modulatory effects were observed in layers 2/3 and 4, but not in layer 5, indicating that the localized modulatory effects resulted from the low-intensity TBS. These findings suggest valuable information about the related methodologies and provide physiological evidence of auditory cortex modulation in mice, which may guide future research aiming to optimize TBS for treating hearing disorders, such as tinnitus.

## Materials and Methods

### Coil parameters

**Figure 1.**
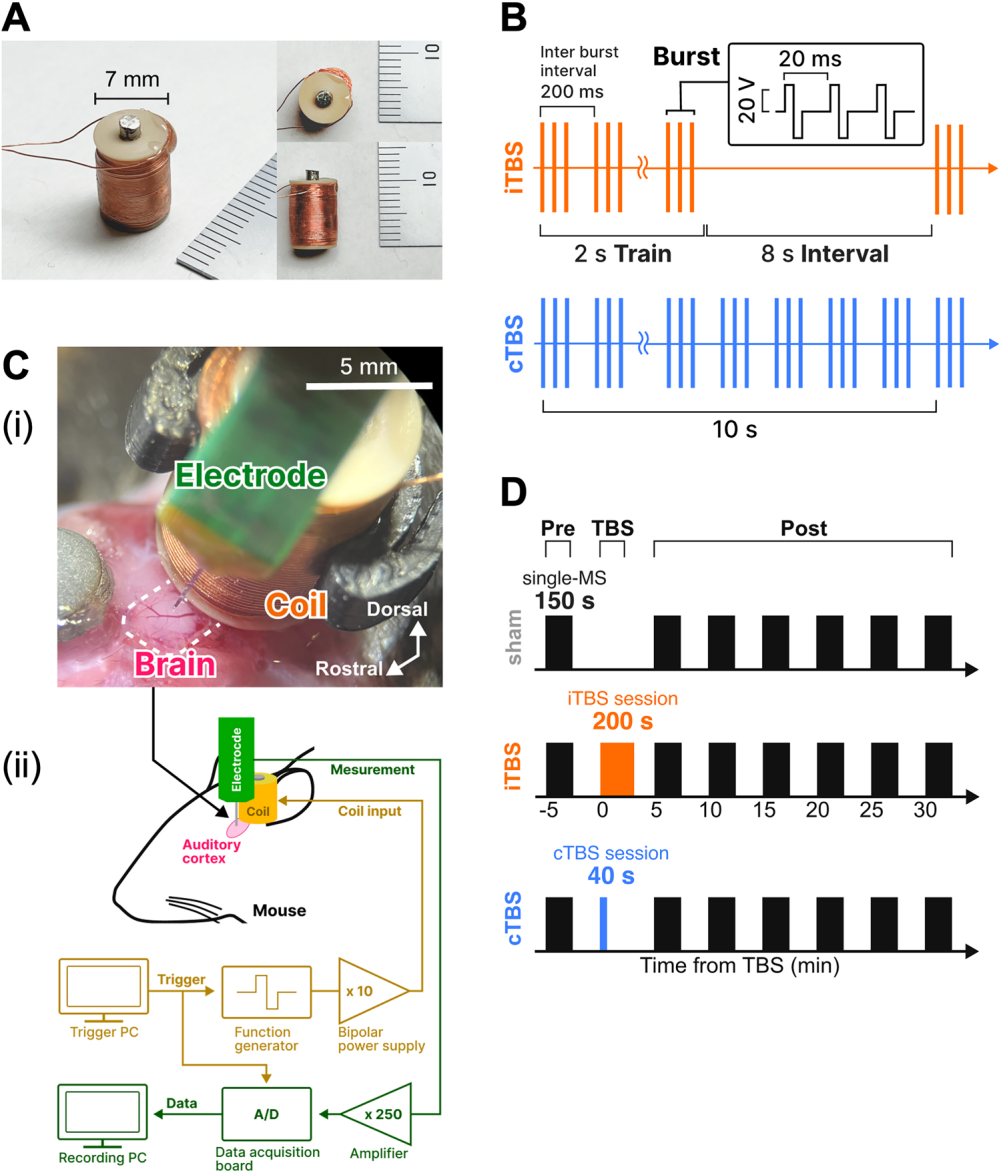
Summary of the experimental methods. ***A***, Constructed millimeter-sized coil with a Permalloy 45 core. ***B***, Stimulation patterns for iTBS and cTBS. Each vertical line represents a single-pulse input to the coil. ***Ci***, Placements of the coil and electrode. The electrode was inserted into the starting point of response observed by FAI (Extended Data Fig. 1-1). ***Cii***, Experimental system for simultaneous stimulation and neural data acquisition. ***D***, Outline of the modulatory experiment. The black bars show the duration of the 0.2 Hz repetitive magnetic stimulation (MS) delivered for MS-evoked activity measurements.

A millimeter-sized coil for magnetic stimulation (MS) was constructed (Shonan Engineering Corporation). The solenoid coil was wound 635 times with 0.16-mm-diameter polyurethane-coated copper wire around a resin bobbin (2 mm inner diameter × 7 mm outer diameter × 10 mm height, Fig. 1*A*). A Permalloy 45 metal rod (2 mm diameter × 12 mm long; The Nilaco Corporation) was inserted into the bobbin hole to serve as the coil core. To prevent winding breakage, epoxy resin was applied to part of the coil.

10.1523/ENEURO.0577-24.2025.f1-1 Figure 1-1 Verification of auditory cortex targeting by flavoprotein autofluorescence imaging. **A,** Auditory cortical activity in response to 4, 8, and 16 kHz tone-burst exposure. Blue arrows indicate the starting point of the response. **B**, Line plots of fluorescent intensity at the starting point of the response (site 1, black line) and a nearby point outside the auditory cortex (site 2, gray line). Download Figure 1-1, TIF file.

### Electromagnetic field simulation

Previous research using rat brains has revealed that modulation occurs in the motor cortex when the induced electric field (*E*) from MS reaches 10 V/m (Tang et al., 2016b). Based on this finding, we set a numerical target of *E* ≥ 10 V/m and used numerical simulations to determine the peak-to-peak voltage (*V*_pp_) required to achieve this target.

To obtain a general estimate of the electric field and magnetic flux density generated in the brain by our coil, we used COMSOL Multiphysics (Ver. 6.1, COMSOL), a general-purpose physics simulation software that is based on the finite element method. The calculations were performed using the application server of a supercomputer system (PRIMERGY CX400/CX2550, Fujitsu).

The coil model geometry was based on the dimensions of the constructed millimeter-sized coil. The coil model was reproduced as a cylinder with an outer diameter of 7 mm, a height of 10 mm, and an inner diameter of 2 mm. It was uniformly wound 635 times by a wire with a diameter of 0.16 mm and a conductivity of 5.998 × 10^7^ S/m. The coil core was modeled as a cylinder with a diameter of 1.8 mm and a height of 12 mm. Materials were set to “copper” for the coil and “nickel steel permalloy oriented” for the coil core. The permeability of the permalloy was set to 1.74 × 10^6^.

The mouse brain geometry was adapted from the three-dimensional (3D) model on Thingiverse (https://www.thingiverse.com/thing:3079327), which is based on the Scalable Brain Atlas (Bakker et al., 2015). The original model had a very fine mesh, resulting in long computation times. The mesh was therefore simplified using the Remesh module of Blender (Ver. 3.1.2, Blender Foundation), an open-source 3D computer graphics software. The Remesh settings were as follows: smooth mode, Octree depth of 4, and scale of 0.9. The surface was then smoothed using the subdivision surface module with the Catmull–Clark method at Level 1.

The brain model imported into the software was rotated 45° along the rostral–caudal axis. The coil model was positioned 0.5 mm above the brain surface, with the end of the coil aligned with the auditory cortex location (9 mm caudal from the rostral end) according to a brain atlas (Allen Institute for Brain Science, 2004). A cylinder representing air was created to cover the coil and the entire brain model, and the “air” material properties were assigned from the material library. All geometries were constructed using the “fine mesh” settings.

A physical model of electromagnetic field analysis (magnetic fields) within the AC/DC module was used. Calculations were made within the frequency domain with a 100 kHz frequency to represent a 10 µs rise time. The input voltages were swept in 5 V intervals from 5 to 25 V (*V*_pp_ = 10–50 V). Boundary conditions included a 0.05 mm gap between the coil and the coil core to prevent errors from domain interference and a 1 mm layer of infinite element domain surrounding the air domain to account for the shape influence on the calculation results. Additionally, the air was assigned a small conductivity (1 × 10^−7^ S/m) to prevent the divergence of calculations. The material parameters used in the simulation are provided in Table 1.

**Table 1.** Material parameters used for electromagnetic field stimulation

Material	Relative permittivity	Relative permeability	Conductivity
Air	1	1	1.0 × 10^−7^
Nickel steel permalloy oriented	1	1.74 × 10^6^	1.74 × 10^−6^
Copper	1	1	5.998 × 10^−7^
Brain tissue	3.52 × 10^3^	1	1.54 × 10^−1^

### Coil property measurements

A biphasic square waveform pulse was used for the voltage input applied to the coil. The waveform was generated by a function generator (WF1947, NF Corporation) and amplified 10-fold using a high-speed bipolar power supply (KIT61380, NF Corporation) before being applied to the coil.

The magnetic flux density of the coil was measured to compare the performance of the constructed coil with the simulation results and previous research. Five Hall effect sensors (SS94A2D, Honeywell) were used to measure the magnetic flux density at *V*_pp_ = 0–60 V of the input waveform. Each Hall effect sensor was positioned directly beneath the coil one at a time, with a 0 mm distance between the sensor and the coil. Magnetic flux density measurements from five Hall effect sensors were averaged to increase measurement reliability. Hall effect sensors were powered by a direct current power supply (PA36-2B, TEXIO) providing 8 V of power. A magnetic flux density measurement system was constructed to synchronize the voltage application to the coil with the timing of magnetic flux density measurements. A digital acquisition (DAQ) system (USB-6343, National Instruments) and a custom-made program created in Python 3.11 were used to control the function generator and to measure the voltage output from the Hall effect sensor. The measurements of the Hall effect sensors were validated by comparing them with those obtained from a calibrated tesla meter (TM-801, Kanetec), where the magnetic flux density induced by a *V*_pp_ = 5 V, 50 Hz sine wave was measured and yielded an observed error of approximately 3%.

Heat generation upon voltage application to the coil was measured using a T-type thermocouple (SCHS1-0 KT-0207C5878, CHINO). Voltage readings from the thermocouple were converted to digital data using an A/D converter (GL100-WL-4VT, Graphtec) and recorded using the associated driver and software (GL100-APS, Graphtec). The coil was fixed in the air using a custom-built holder and a micromanipulator (SM-15L, Narishige), while the thermocouple tip was precisely positioned at the bottom of the coil using another micromanipulator. Temperature changes were measured for the 0.2 Hz repetitive MS (rMS), cTBS, and iTBS patterns. TBS was administered as bursts of three biphasic rectangular pulses at 20 ms intervals (50 Hz), repeated with a 200 ms interburst interval (5 Hz; Fig. 1*B*). In the cTBS condition, the burst waveform was continuously presented for 40 s (600 pulses in total). In the iTBS condition, the burst waveform was presented for 2 s within every 10 s interval, over a period of 200 s (600 pulses in total). The temperature was recorded for 300 s at each voltage level (*V*_pp_ = 10, 20, 30, 40, and 50 V). Additionally, temperature changes on the brain surface during each stimulation pattern (*V*_pp_ = 40 V) were measured by placing the coil and thermocouple directly on the mouse's brain surface.

Sound emission from the coil during MS at *V*_pp_ = 40 V was measured. A microphone (Type 4939-L-002, Brüel and Kjær) was placed 2.5 mm away from the coil to record sound pressure changes. The voltage measured from the microphone was amplified using a measuring amplifier (Type 2636, Brüel and Kjær) and recorded using a DAQ system (USB-6343) at a sampling frequency of 500 kHz. Recordings were made in a soundproof room with the door closed. To eliminate MS artifacts, baseline recordings were obtained by placing a microscope glass slide between the microphone and the coil (Extended Data Fig. 2-1A). Sound recordings were made over 100 trials for each condition (with and without the glass slide), and the waveforms were averaged across the trials. The sound waveform without the MS artifact was obtained by subtracting the baseline waveform from the waveform recorded without the acrylic board (Extended Data Fig. 2-1*B*–*D*).

### Surgical procedures

All animal procedures were performed in accordance with the Hokkaido University Animal Care Committee's regulations.

Fifteen C57BL/6J mice aged 7–12 weeks (five males and 10 females) were used. All mice were housed in plastic cages with free access to water and food. The mice were segregated by sex and kept in cages with no more than five mice per cage. The room temperature was maintained at 20–22°C, and the light cycle was set to a 12 h light/dark schedule to reproduce day and night conditions.

Mice were anesthetized by urethane (1.5 g/kg body weight, Wako). To reduce sensory input other than auditory information, the eyelids were closed with instant adhesive to block vision, and the whiskers were cut to reduce somatosensory input. Hair on the top of the head and the left temporal area was shaved, and topical anesthesia (Xylocaine Jelly 2%, Sandoz Pharma K.K.) was applied to the scalp. The scalp and periosteum were then incised to expose the parietal portion of the skull. A custom-built fixation device was attached to the parietal bone using instant adhesive and dental cement. The fixation device was then bolted to a custom-made fixation stand to stabilize the cranial position of the mice.

The temporal muscle was removed to expose the skull near the auditory cortex, and a craniotomy was performed using a dental drill. The dura mater was then carefully removed using a disposable needle and precision scissors (8-cm-long Vannas Scissors, CVD 14122, World Precision Instruments) to expose the brain. A saline solution was periodically applied to prevent desiccation of the brain surface.

To identify the auditory cortex among the surgically exposed brain regions, flavoprotein autofluorescence imaging (FAI) was used (Extended Data Fig. 1-1). FAI was conducted using an upright microscope (THT, Brainvision) equipped with a 1× objective lens (numerical aperture 0.23, Plan Apo 1×, Leica Microsystems) and a complementary metal oxide semiconductor (CMOS) imaging system (MiCAM02, Brainvision). Excitation light was provided by a blue light-emitting diode light source (LEX2-B, Brainvision), which passed through a bandpass filter (wavelength, 466 ± 20 nm; No. 86-352, Edmund Optics) and was reflected 90° by a dichroic mirror (wavelength, 509 nm; FF509-Fdi01-15x36, Semrock) onto the brain. The autofluorescence of flavoproteins generated in the brain passed through the dichroic mirror and an absorbance filter (wavelength, 525 ± 45 nm; Edmund Optics) before being captured by the CMOS camera. The imaging frequency was set to 20 frames/s, and fluorescence images were recorded for 5 s, centered around the sound stimulation timing.

For each FAI trial, tone bursts of 80 dB sound pressure level (SPL) with frequencies of 4, 8, and 16 kHz and a duration of 100 ms were used as the stimulus sound. Each frequency was presented 10 times, once every 8 s. The captured images were averaged using a dedicated software (BV Ana, Brainvision). The averaged images were further processed using a 5 × 5 pixel moving average spatial filter and a five-frame moving average temporal filter. The resulting fluorescent images were played back as an animation, and the location at which the response propagation began was identified as the primary auditory cortex, which was then used as the insertion point for the electrode. The electrode was inserted at the site most responsive to 8 kHz sound stimulation, provided a response was detected in the FAI. If the response to the 8 kHz stimulation was weak, sites responsive to other frequencies (i.e., 4 or 16 kHz) were used instead. Consequently, the insertion site remained consistent across animals, located approximately 2.8 mm posterior and 4.8 mm lateral to the bregma.

### Neural data recording

Extracellular recordings were conducted by inserting a multielectrode array (A1x16-5mm-50-177, NeuroNexus) into the auditory cortex region (identified using FAI) of anesthetized mice. The multielectrode array was secured with a metal rod. Using a micromanipulator (SM-15L, Narishige), the electrode was positioned so that its tip touched the surface of the auditory cortex, and it was then inserted 800 µm. Next, the millimeter-sized stimulation coil was placed on the caudal side of the exposed brain surface, approximately 0.5 mm caudal to the insertion point (Fig. 1*Ci*). To prevent the brain surface from drying out, agarose gel (0.45%, Kanto Chemical) was applied. The coil was secured using a micromanipulator (SM-15L, Narishige) and a custom-built holder that was fabricated using a 3D printer. An Ag/AgCl reference electrode was placed approximately 1 mm rostral to the exposed area.

Neural activity was recorded in a soundproof room. Signals obtained from the multielectrode array were amplified in two stages using two amplifiers, for a total gain of 250×, and were recorded at a sampling frequency of 40 kHz using a data acquisition board (OmniPlex, Plexon). The timing of the MS was recorded by capturing the trigger output from the DAQ with the data acquisition board (Fig. 1*Cii*).

The obtained neural signals were analyzed by filtering them into local field potentials (LFPs) and MUAs. A 50 Hz low-pass filter was applied to focus on the frequencies below the gamma range and to remove artifacts caused by MS. MSEPs were obtained by cropping the LFP waveforms around the MS timestamp and were averaged across trials for peak detection. Because LFPs are generated primarily by synaptic currents entering a population of neurons (current sinks), the negative peak amplitude serves as an indicator of local synaptic input intensity (Herreras, 2016). Therefore, we obtained the negative peak within 100 ms from the stimulus onset in the average MSEP waveform and used the negative peak amplitude for analysis. MSEP waveforms from layers 2/3, 4, and 5 (one channel per layer, three layers per animal) were included for statistical analysis.

A 600–8,000 Hz bandpass filter was then applied to the raw waveforms to extract the high-frequency components, and spikes were detected using a custom-made program written in Python 3.11. For spike detection, the spike detection algorithm from the spike sorting software Offline Sorter (Plexon) was used. First, the standard deviation of the entire recorded waveform $\left(\sigma_{\mathrm{full}}\right)$ was calculated. Next, the noise portion within $2.7\times \sigma_{\mathrm{full}}$ was extracted, and its standard deviation $\left(\sigma_{\mathrm{noise}}\right)$ was calculated. A threshold of $-3\times \sigma_{\mathrm{noise}}$ was then set, and the point at which the waveform crossed this threshold from above was identified as the spike timestamp. The waveform around this timestamp was detected as the spike waveform. The waveforms were cropped from −200 µs before the spike timestamp, with a width of 800 µs. Finally, spikes with an amplitude greater than $20\times \sigma_{\mathrm{noise}}$ and those occurring within 5 ms after MS were considered artifacts and were excluded.

### Event-related activity recording

Neural responses to sound and MS were measured. For each condition, stimuli were delivered at a frequency of 0.2 Hz (interstimulus interval of 5 s) with 30 repetitions.

To confirm that the electrode was inserted in the auditory cortex, neural responses to sound stimuli were recorded. An 80 dB SPL, 4 kHz tone burst with a duration of 100 ms was used as the sound stimulation. Signal generation and timing control for the stimuli were performed using a custom-made Python program, which outputs sound waveforms and stimulation triggers from a DAQ system (NI-6259, National Instruments). The sound waveforms were then amplified by an amplifier (SA1, Tucker-Davis Technologies) and supplied to a speaker (MF-1, Tucker-Davis Technologies).

We then recorded neural responses to single-pulse MS (single-MS). The coil's input signal was a biphasic pulse with *V*_pp_ = 40 V, which was generated by a function generator and bipolar power supply, as described in the previous section. The timing of the MS was controlled using the trigger output from the DAQ. Neural responses to single-MS were measured under two conditions: regular MS and MS postmortem. The postmortem condition was used to confirm that the neural responses were not artifacts of MS; the coil was positioned in the same way as during regular MS. The mice were killed by administering an overdose of anesthesia.

### Modulation induction experiment

A modulation induction experiment was conducted to evaluate whether TBS using our millimeter-sized coil was able to modulate auditory cortical functions in mice. Both iTBS, known for its facilitatory effect, and cTBS, known for its inhibitory effect, were applied to the mouse auditory cortex. Neural responses to single-MS (one stimulus every 5 s for a total of 30 trials over 150 s) were measured every 5 min before and after TBS (pre- and post-TBS phases, Fig. 1*D*). For both single-MS and TBS, a biphasic rectangular waveform at *V*_pp_ = 40 V was used. The input signal to the coil was applied using the same method as described in the previous section.

Neural activity during TBS delivery was analyzed to detect potential changes in the response patterns. The negative peak amplitude of the MSEP for each TBS burst was then extracted for analysis. To assess the temporal changes in MSEP amplitudes during TBS, the bursts were segmented into 10 equal sections (each comprising 20 ms). The mean MSEP waveform and corresponding negative peak amplitude were computed for each section. Statistical analyses were then performed to evaluate the temporal changes in MSEP amplitudes across these sections.

iTBS consisted of 10 bursts of triplet pulses within a single 2 s train. To examine how the MSEP amplitude changed within the iTBS train, we grouped the TBS bursts by their position (*n*th burst) within each train (from the first to the 10th). The negative peak amplitude of the MSEP was then computed for each burst number and compared. No similar analysis was performed for cTBS, as it is continuous and does not consist of discrete bursts.

To identify the modulatory effects induced by TBS, neural responses were analyzed in both the pre- and post-TBS phases. The changes of MSEPs were assessed by comparing the negative peak amplitudes across three conditions: (1) cTBS, (2) iTBS, and (3) sham stimulation (*V*_pp_ = 0 V). For MUA, peristimulus time histograms (PSTHs) were generated using 5 ms bins to count spikes before and after single-MS. Next, the number of spikes occurring 5–50 ms after single-MS was computed by subtracting the baseline spike count (the number of spikes occurring 5–50 ms before stimulation) and was compared among the three conditions. Additionally, the negative peaks of average spike waveforms occurring 5–50 ms poststimulation were calculated and subjected to statistical analysis.

Statistical analyses were conducted using the Kruskal–Wallis test to assess significant differences among multiple conditions. When significant differences were detected, post hoc comparisons were performed using the Steel–Dwass test. The level of significance was set to 5%.

## Results

### Measured coil properties

**Figure 2.**
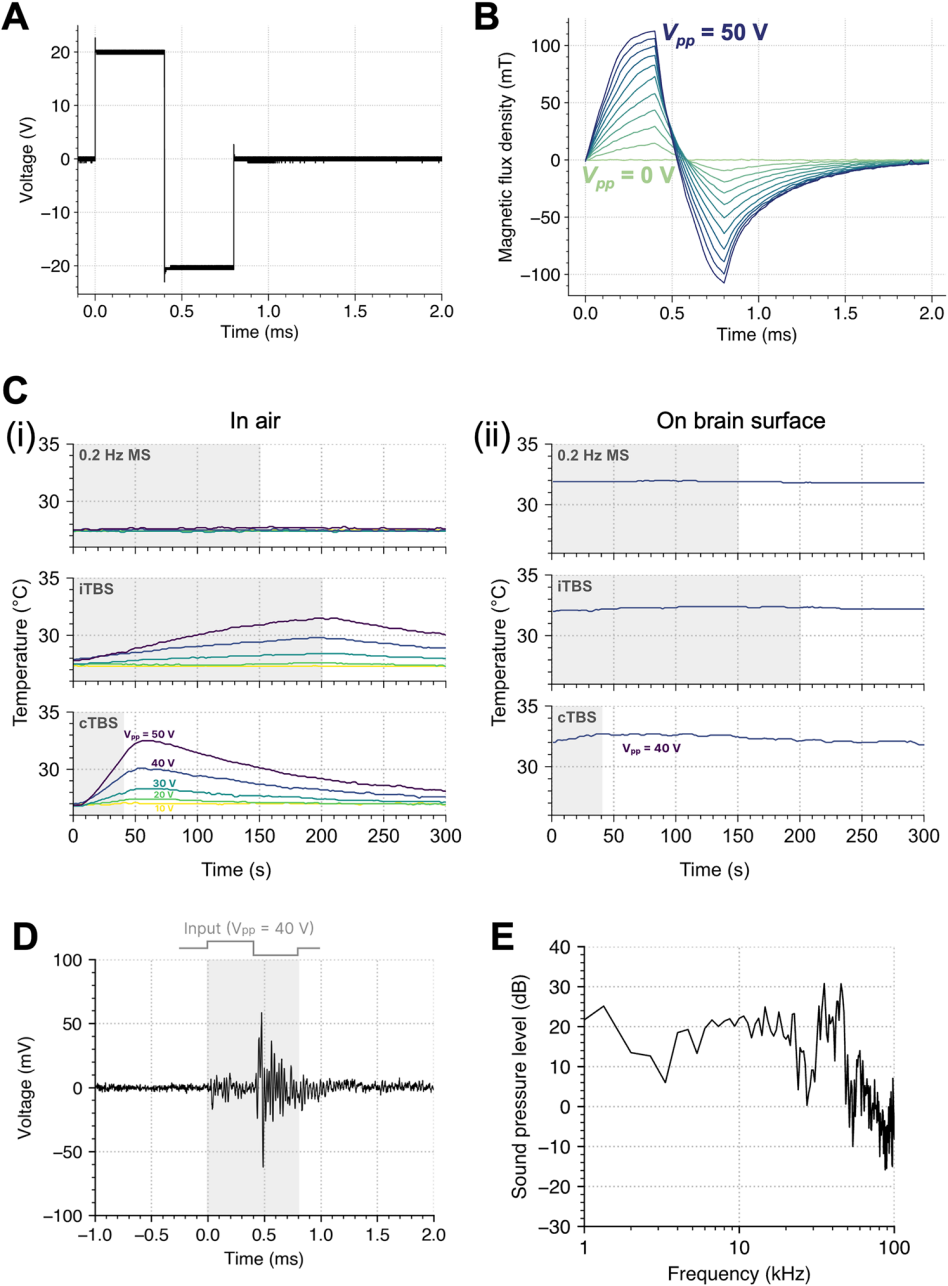
Custom-made coil properties and magnetic stimulation (MS) effects associated with heat generation and acoustic noise. ***A***, Waveform of the bipolar pulse input to the coil. ***B***, Magnetic flux density for each input voltage. Each line corresponds to a different input voltage (*V*_pp_ = 0, 5, …, 50 V). ***C***, Change in temperature at the bottom of the coil (***i***) in air and (***ii***) on the brain surface for each stimulation pattern. Each line corresponds to a different input voltage (*V*_pp_ = 20, 30, 40, 50 V). ***D***, Waveform of the sound recorded from the coil at *V*_pp_ = 40. Gray shading represents the time at which voltage was applied. Raw responses with and without the glass slide are provided in Extended Data Figure 2-1. ***E***, Frequency spectrum of the sound emitted from our coil. The sound waveform shown in Figure 2*D* (0–1.5 ms) was transformed into frequency components using fast Fourier transformation. The maximum sound pressure level (SPL) was 30.8 dB SPL at 35 kHz.

Because electric fields are challenging to measure, magnetic flux density is used as a more accessible metric for assessing the intensity of MS, thus allowing the comparison of stimulation intensities across different studies. Figure 2*A* illustrates the waveform of the biphasic pulse input applied to the coil, with a peak-to-peak voltage (*V*_pp_) of 40 V. The waveform of the magnetic flux density measured using the Hall effect sensor is shown in Figure 2*B*. In accordance with the biphasic pulse input, both positive and negative magnetic flux densities were observed. The positive peak magnetic flux densities were 1.1 ± 0.04 mT at *V*_pp_ = 0 V, 14.6 ± 0.8 mT at *V*_pp_ = 5 V, 29.4 ± 1.4 mT at *V*_pp_ = 10 V, 43.6 ± 2.3 mT at *V*_pp_ = 15 V, 58.0 ± 2.9 mT at *V*_pp_ = 20 V, 73.0 ± 3.5 mT at *V*_pp_ = 25 V, 83.0 ± 4.0 mT at *V*_pp_ = 30 V, 91.4 ± 4.4 mT at *V*_pp_ = 35 V, 99.6 ± 5.1 mT at *V*_pp_ = 40 V, 106.3 ± 5.0 mT at *V*_pp_ = 45 V, and 112.9 ± 5.2 mT at *V*_pp_ = 50 V.

10.1523/ENEURO.0577-24.2025.f2-1 Figure 2-1 Sound emission measurements from the coil. **A**, Placement of the coil and microphone with and without the glass slide. **B**, Average waveform of the recorded signal with a microscope glass slide inserted between the coil and the microphone, **C**, without the glass slide, and D, the speculated sound waveform obtained by subtracting the waveform recorded with the glass slide from that without the glass slide. The result for 40 V was previously shown in Fig. 2D. **E**, Hearing thresholds of the mice in our lab (*n* = 5, 6–10 weeks old). The hearing threshold was defined as the lowest tone-burst (100 ms, 2–32 kHz) intensity evoking an auditory brainstem response (ABR). The average ABR waveform of 512 trials was used for the hearing threshold detection. Initial hearing thresholds are shown in blue. Hearing thresholds recorded 2 weeks after the initial recording (8–12 weeks old) are shown in red. Error bars indicate the standard error. The gray line indicates the sound pressure level of the sound emitted from our coil. **F**, Representative LFP waveforms in response to the MS sound (Fig. 2D) played from the speaker. The MS sound was calibrated to achieve a maximum sound pressure level of 30.8 dB in its frequency components, ensuring consistency with Fig. 2E. Plot formats are the same as in Fig. 4. **G**. Comparison of the peak amplitude across coil noise, sham (no sound), and tone-burst conditions (9 recording points per condition, 3 animals × 3 channels). **p < 0.01 (Kruskal–Wallis test followed by post hoc Steel–Dwass test) ^h1, h2^. Download Figure 2-1, TIF file.

Temperature is a crucial factor that modulates neuronal excitability. We therefore measured the temperature changes at the bottom of the coil while fixed in air during single-MS and TBS. The temperature changes during MS sessions are shown in Figure 2*C*. Single-MS resulted in almost no temperature change, with peak changes of 0°C at *V*_pp_ = 10 V, 0.1°C at *V*_pp_ = 20 V, 0.1°C at *V*_pp_ = 30 V, 0.4°C at *V*_pp_ = 40 V, and 0.3°C at *V*_pp_ = 50 V. For iTBS, the peak temperature changes were 0.07°C at *V*_pp_ = 10 V, 0.1°C at *V*_pp_ = 20 V, 0.9°C at *V*_pp_ = 30 V, 1.9°C at *V*_pp_ = 40 V, and 3.7°C at *V*_pp_ = 50 V. For cTBS, the peak temperature changes were 0.2°C at *V*_pp_ = 10 V, 0.5°C at *V*_pp_ = 20 V, 1.5°C at *V*_pp_ = 30 V, 3.1°C at *V*_pp_ = 40 V, and 5.7°C at *V*_pp_ = 50 V. Additionally, the temperature changes on the brain surface were measured while applying single-MS and TBS with an input voltage of *V*_pp_ = 40 V, which resulted in a maximum increase of 0.1°C during single-MS, 0.4°C during iTBS, and 0.7°C during cTBS.

When a current is applied to a coil, sound can be generated because of the mechanical vibrations of the windings produced by magnetic fields. To assess the amount of sound emitted from the coil during MS, we recorded the sound using a microphone. Figure 2*D* shows the sound waveform recorded from the coil, and Figure 2*E* shows the frequency spectrum of the recorded sound. The maximum observed SPL was 30.79 dB SPL at 35 kHz.

In summary, magnetic flux density increased with *V*_pp_, and sound emissions peaked at 30.8 dB SPL. Single-MS sessions caused very minimal temperature changes, whereas iTBS and cTBS led to small temperature rises. Together, these findings demonstrate that single-MS had minimal thermal and acoustic impact.

### Simulated coil properties

**Figure 3.**
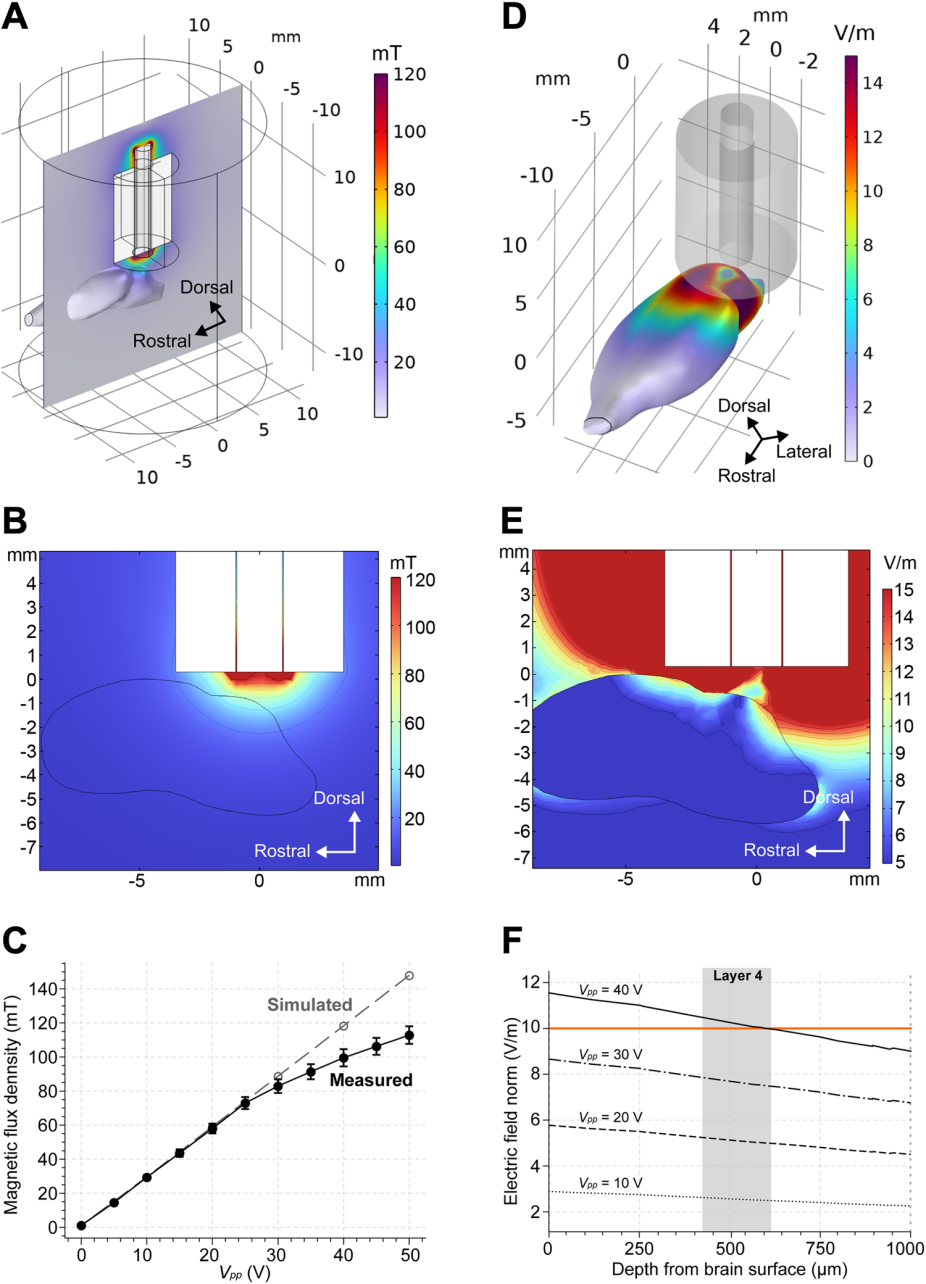
Simulation results. ***A***, Magnetic flux density in three-dimensional space. ***B***, Magnetic flux density norm in the brain. The cross section shown in (***A***) was used, which passes through the center of the coil and is parallel to the rostral direction of the brain. ***C***, Comparison of recorded and simulated magnetic flux density. ***D***, Electric field in three-dimensional space. A top-down view of (***D****)* is provided in Extended Data Figure 3-1. ***E***, Electric field norm in the brain. The same cross section as (***B***) was used. ***F***, Change of electric field by depth in the brain for each voltage input. *V*_pp_ = 40 surpassed our numerical target of *E* ≥ 10 (orange line).

Electromagnetic fields were estimated using numerical simulations to determine the appropriate stimulation intensity for the modulation experiment. To ensure that the simulation conditions accurately reflected the actual measurements, the magnetic flux density was subsequently estimated and compared with the measured data. Figure 3*A* illustrates the distribution of magnetic flux density in 3D space, and Figure 3*B* shows the cross section indicated in Figure 3*A*. In Figure 3*C*, the estimated magnetic flux density values are compared with the actual measurements obtained using the Hall effect sensor. The estimated magnetic flux density values were 29.6 mT at *V*_pp_ = 10 V, 59.2 mT at *V*_pp_ = 20 V, 88.8 mT at *V*_pp_ = 30 V, 118.3 mT at *V*_pp_ = 40 V, and 147.9 mT at *V*_pp_ = 50 V.

10.1523/ENEURO.0577-24.2025.f3-1 Figure 3-1 Top-down view of Fig. 3D, showing the spatial distribution of the simulated electric field (V/m) induced by the coil. The color scale represents the electric field strength, with warmer colors indicating higher intensities. Download Figure 3-1, TIF file.

The electric fields were then estimated to determine the stimulation intensity that was required to achieve the numerical target of *E* ≥ 10 V/m. Figure 3*D* illustrates the 3D distribution of the electric field, whereas Figure 3*E* shows the 2D distribution of the electric field in the same cross section as in Figure 3*B*. In Figure 3*F*, the estimated electric fields at various depths from the brain surface are shown for each input voltage. At *V*_pp_ = 40 V, the electric field reached our numerical target within depths ranging from 0 to 600 µm, which covers layers 1–4 of the cortex.

### LFP responses to sound and single-MS

**Figure 4.**
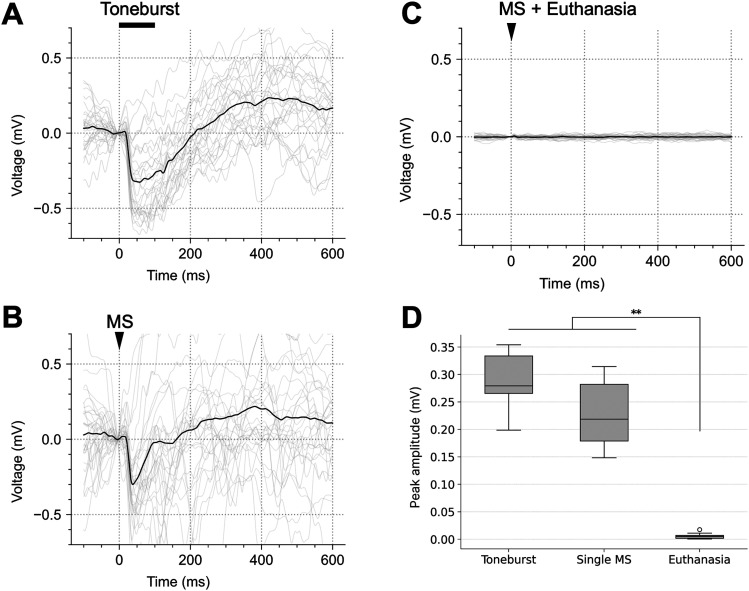
Representative LFP waveforms in response to (***A***) tone-burst stimulation, (***B***) magnetic stimulation (MS), and (***C***) MS postmortem. For each plot, the gray lines represent raw LFP plots (30 trials), and the black lines represent the mean LFP waveform. LFP waveforms were obtained by applying a 50 Hz low-pass filter to the wideband signal. ***D***, Comparison of the peak amplitude across tone-burst, single-pulse MS (single-MS), and postmortem conditions (9 recording points, 3 animals × 3 channels). **p < 0.01 (Kruskal–Wallis test followed by post hoc Steel–Dwass test). The methods and results of statistical tests used in this study are listed in Extended Data Table 4-1.

To confirm that the recording site was in the auditory cortex, responses to tone-burst sounds were recorded. Additionally, to confirm that MS induces neural activity, neural responses to single-MS were recorded. In Figure 4, typical LFP waveforms in response to tone-burst (Fig. 4*A*), single-MS (Fig. 4*B*), and single-MS postmortem (Fig. 4*C*) are shown. Some waveforms exhibited high variability across trials due to strong spontaneous activity, but the average waveform remained stable and representative (Fig. 4*B*).

10.1523/ENEURO.0577-24.2025.t4-1 Table 4-1 List of the methods and results of statistical tests used in this study. Significant *p*-values (<0.05) are indicated in bold text. Download Table 4-1, XLSX file.

The LFP responses to tone burst and single-MS had significantly larger peak amplitudes compared with the single-MS postmortem condition (Fig. 4*D*; tone burst, *p* = 0.001; single-MS, *p* = 0.001; Extended Data Table 4-1, a1 and a2).

The LFP responses to tone-burst sounds confirmed that the recording site was indeed in the auditory cortex. Moreover, the finding of significantly larger peak amplitudes in single-MS recordings than in postmortem single-MS recordings indicates that the single-MS responses were not MS artifacts.

### MSEPs during TBS delivery

**Figure 5.**
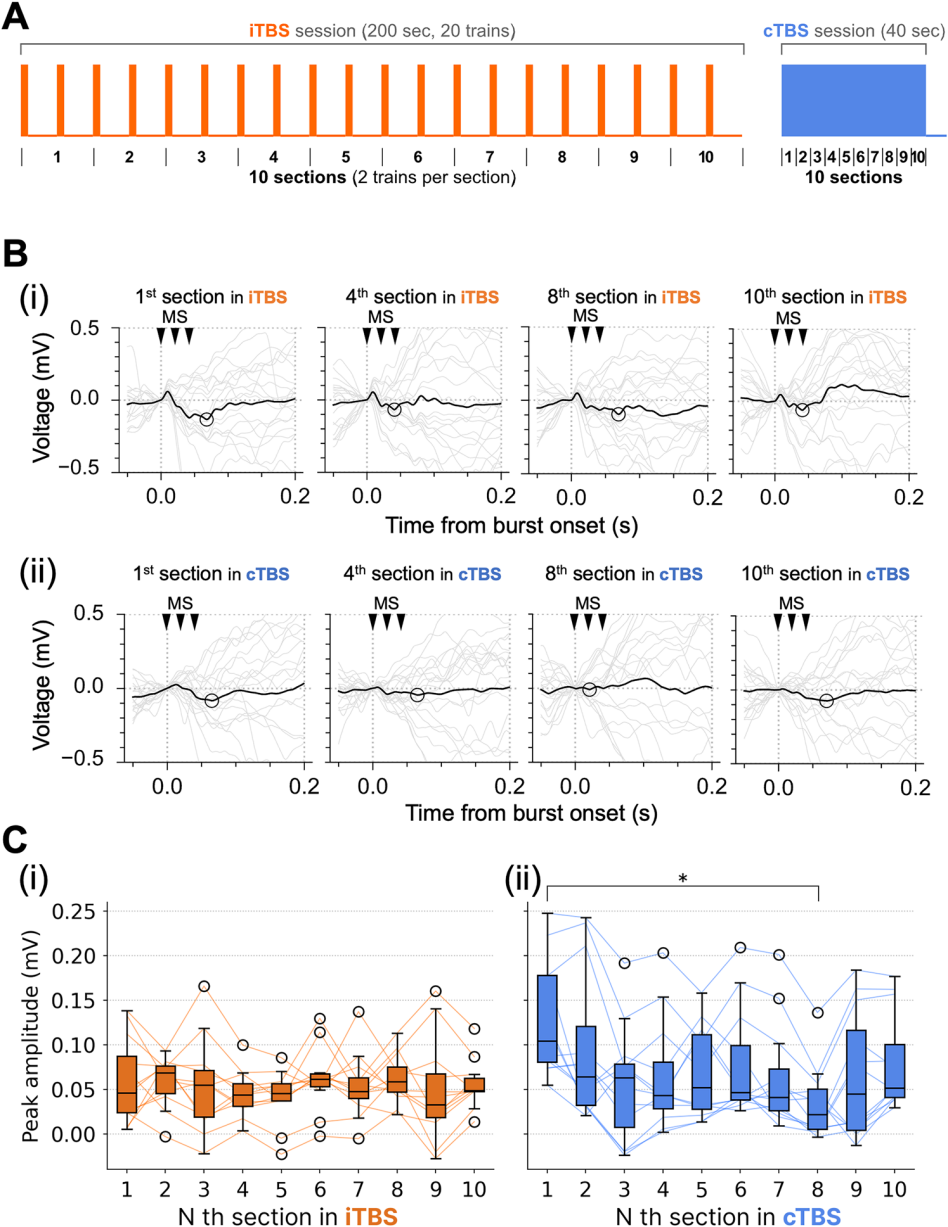
Analysis of MSEPs during iTBS/cTBS delivery. ***A***, Definition of the sections in iTBS/cTBS sessions. Each section in an iTBS session was 20 s in duration and contained two trains (20 bursts). Each section in a cTBS session was 4 s in duration (20 bursts). ***B***, Representative average MSEP waveforms for the *n*th section in the (***i***) iTBS and (***ii***) cTBS sessions. MSEP waveforms were obtained by applying a 50 Hz low-pass filter to get rid of the magnetic stimulation (MS) artifacts. The detected peaks are indicated by black circles. Other plot formats are the same as in Figure 4. Latencies of the detected peaks are shown in Extended Data Figure 5-1. ***C***, Peak MSEP amplitudes for each section in the (***i***) iTBS and (***ii***) cTBS sessions. The line plots show the peak amplitude changes measured from a single recording point (12 recording points in total from 4 animals). *p < 0.05 (Kruskal–Wallis test followed by post hoc Dunn test).

To explore changes in neural activity during TBS delivery, MSEP peak amplitudes in response to iTBS and cTBS were measured. To observe how MSEP amplitudes change over time, TBS bursts were divided into 10 sections, with 20 bursts in each section (Fig. 5*A*). Typical MSEP waveforms during the iTBS sections are shown in Figure 5*Bi*. For iTBS, there were relatively consistent peak amplitudes across the sections, with medians ranging from 0.03 to 0.07 mV. There were also no significant changes in MSEP amplitudes for iTBS across the sections (Fig. 5*Ci*, *p* = 0.7, Kruskal–Wallis test; Extended Data Table 4-1, b). Typical MSEP waveforms during the cTBS sections are shown in Figure 5*Bii*. For cTBS, the peak amplitudes varied greatly across sections, ranging from −0.02 to 0.25 mV. Furthermore, a downward trend in peak amplitudes was observed, with the median decreasing from 0.1 mV in the first section to 0.02 mV in the eighth section. Consistent with this observation, the first section had a significantly larger MSEP amplitude than the eighth section (Fig. 5*Cii*, *p* = 0.0046, Steel–Dwass test; Extended Data Table 4-1, c1 and c2).

10.1523/ENEURO.0577-24.2025.f5-1 Figure 5-1 Histograms of peak latencies detected from average MSEP waveforms during iTBS and cTBS. Data from all sections in iTBS/cTBS (see Fig. 5 for details) were included (10 sections × 4 animals × 3 channels). The peaks had similar latencies, around 40 and 70 ms for iTBS and 60 ms for cTBS. Download Figure 5-1, TIF file.

**Figure 6.**
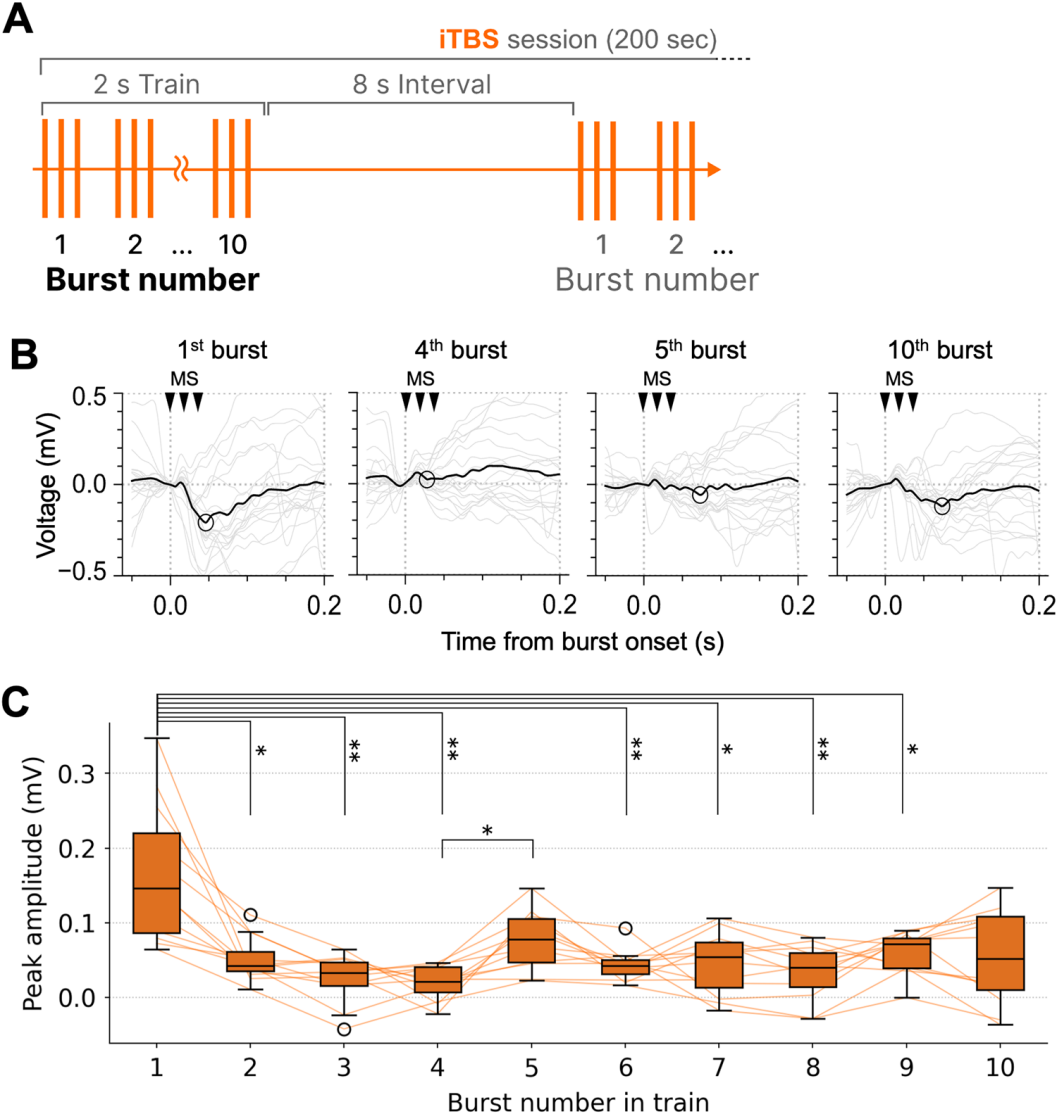
Analysis of MSEPs for each burst in iTBS trains. ***A***, Definition of burst numbers and trains in an iTBS session. ***B***, Representative average MSEP waveforms for the *n*th burst in iTBS trains. Filtering and plot formats are the same as in Figure 5*B*. ***C***, Peak MSEP amplitudes for each burst in iTBS trains. *p < 0.05, **p < 0.01 (Kruskal–Wallis test followed by post hoc Steel–Dwass test).

To examine how MSEP amplitudes change within each iTBS train (i.e., 10 bursts delivered within 2 s), the TBS bursts were grouped by their position (from the 1st to the 10th burst) within each iTBS train (Fig. 6*A*). Typical MSEP waveforms are shown in Figure 6*B*. The average peak amplitude of the first burst in the iTBS trains (median ≈ 0.15 mV) was significantly larger than that of most other bursts (Fig. 6*C*; 2nd, *p* = 0.012; 3rd, *p* = 0.0013; 4th, *p* = 0.0013; 6th, *p* = 0.003; 7th, *p* = 0.016; 8th, *p* = 0.005; 9th, *p* = 0.048; Steel–Dwass test; Extended Data Table 4-1, d1 and d2). Interestingly, the fifth burst had a slightly higher amplitude than the surrounding bursts and a significantly higher amplitude than the fourth burst (*p* = 0.019).

In summary, iTBS maintained consistent MSEP peak amplitudes across sections, whereas cTBS had lower MSEP amplitudes over time. Additionally, the first burst in iTBS trains exhibited higher amplitudes than later bursts. Together, these findings highlight the differing effects of iTBS and cTBS on neural activity during TBS delivery.

### MSEP changes after TBS delivery

**Figure 7.**
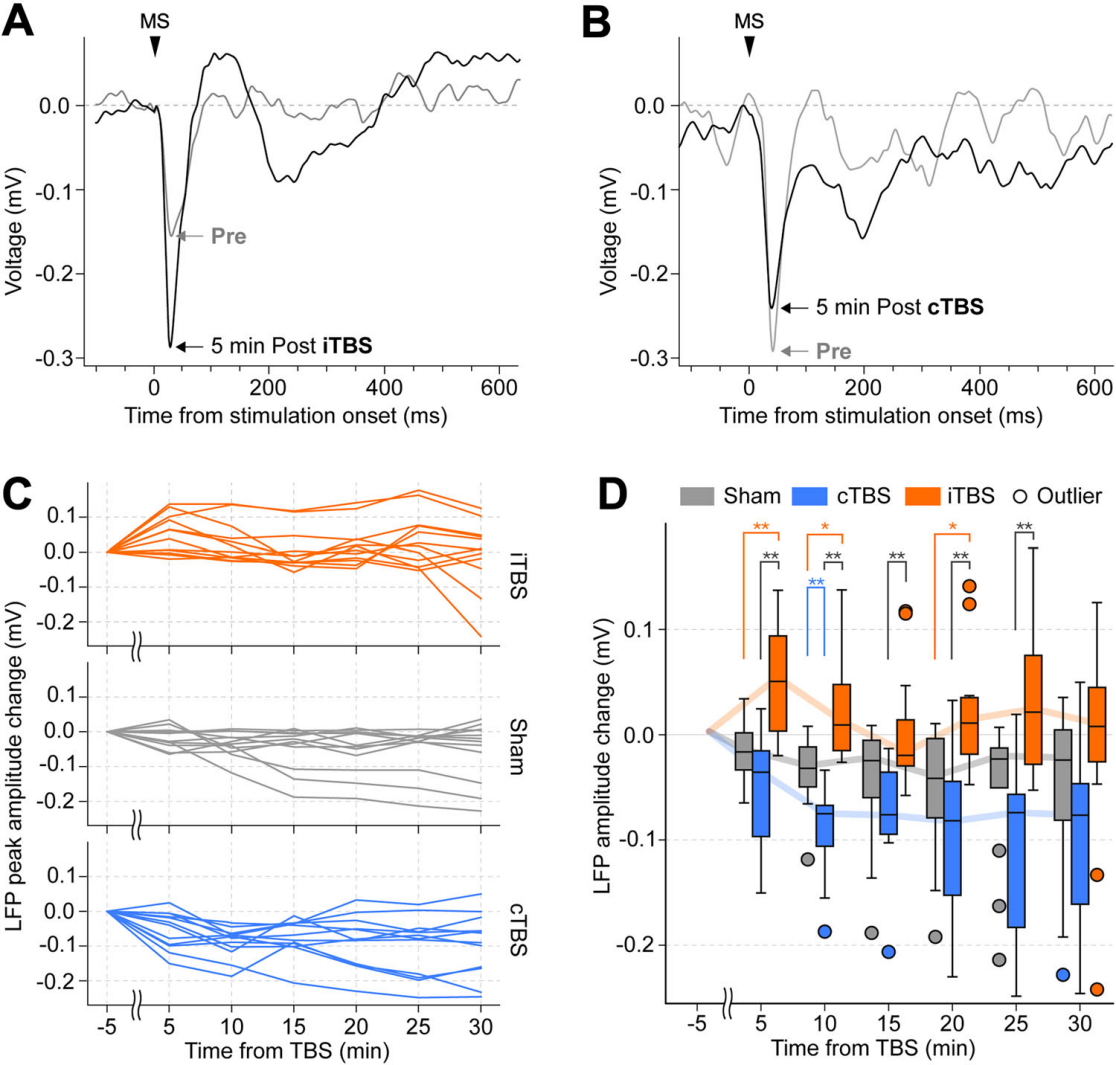
Analysis of MSEPs before and after iTBS/cTBS delivery. ***A***, Representative average MSEP waveforms 5 min before (pre, gray line) and after (post, black line) the iTBS and (***B***) cTBS sessions. ***C***, Changes in peak MSEP amplitudes after iTBS/cTBS delivery. Each line corresponds to the peak amplitude changes measured from a single recording point (12 recording points in total, 4 animals × 3 channels). ***D***, Box plots of peak amplitude changes. The line plots trace the median amplitude changes. Sham (gray), cTBS (blue), and iTBS (orange) groups are shown, with outliers indicated by circles. *p < 0.05, **p < 0.01 (Kruskal–Wallis test followed by post hoc Steel–Dwass test).

TBS has bidirectional modulatory effects; however, this has primarily been investigated in studies targeting the motor cortex. To determine whether such modulation can also be induced in the rodent auditory cortex, we analyzed changes in neural activity across the auditory cortex before and after TBS. To do this, we analyzed changes in MSEPs before and after TBS. Typical average MSEP waveforms before and after TBS delivery are shown in Figure 7, *A* and *B*, where an example of changes in peak amplitudes can be observed: the peak amplitude increased 5 min after iTBS and decreased 5 min after cTBS. Although some MSEP waveforms (Fig. 5*Bi*) exhibited a small positive peak at response onset (0–10 ms), this response component was not consistently observed across mice.

The changes in MSEP peak amplitudes from 5 to 30 min after TBS are shown in Figure 7*C*. When comparing peak amplitudes from 5 to 30 min after TBS with those of the sham group, significant increases were observed at 5, 10, and 20 min after iTBS (5 min, *p* = 0.008; 10 min, *p* = 0.013; 20 min, *p* = 0.025), and a significant decrease was observed 10 min after cTBS (*p* = 0.006; Extended Data Table 4-1, e1 and e2). Additionally, when comparing peak amplitudes between iTBS and cTBS, peak amplitudes were significantly higher for iTBS from 5 to 25 min after TBS (5 min, *p* = 0.001; 10 min, *p* = 0.001; 15 min, *p* = 0.002; 20 min, *p* = 0.003; 25 min, *p* = 0.002; Extended Data Table 4-1, e1 and e2).

In summary, TBS effectively modulated neural activity in the rodent auditory cortex. iTBS resulted in significant increases in MSEP peak amplitudes at 5, 10, and 20 min post-iTBS, whereas cTBS led to a significant decrease at 10 min. These results demonstrate the bidirectional modulatory effects of TBS on neural activity within the auditory cortex.

### MUA changes after TBS delivery

**Figure 8.**
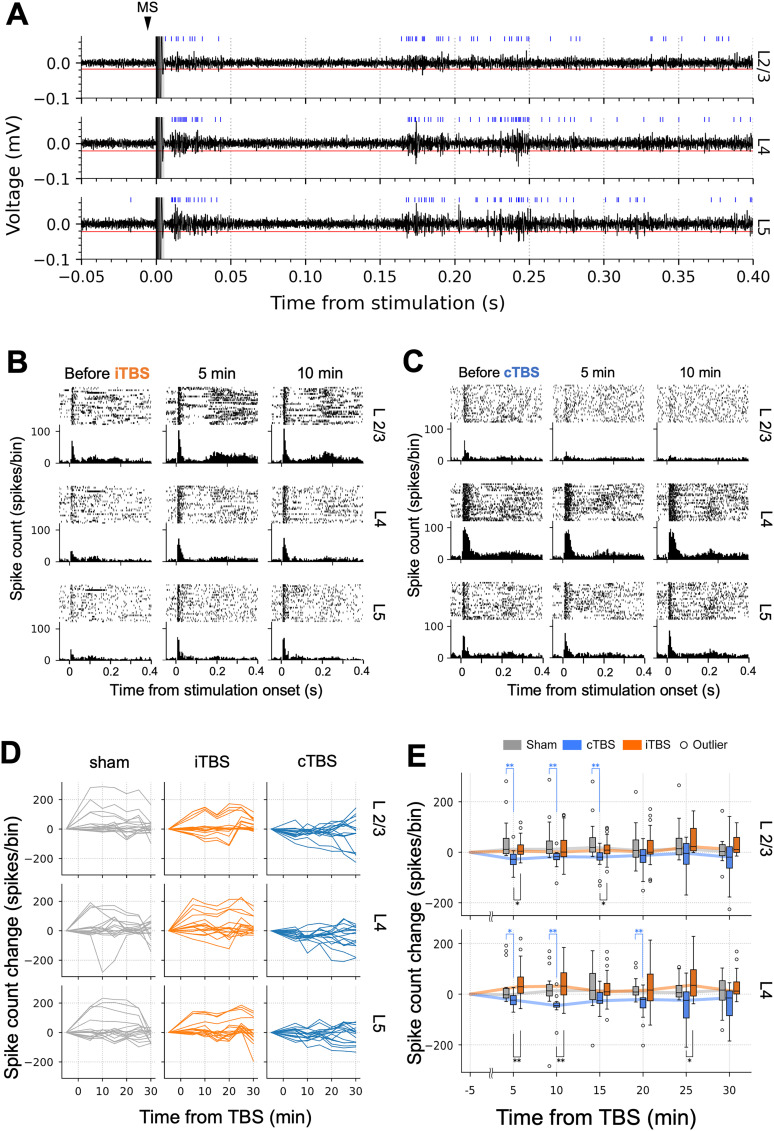
Analysis of multiunit activity (MUA) before and after iTBS/cTBS delivery. ***A***, Representative waveforms of high-pass filtered neural activity. Recordings from layers 2/3, 4, and 5 (L2/3, L4, and L5, respectively) are shown. The red horizontal lines indicate the thresholds for spike detection. The blue vertical lines indicate the timestamps of detected spikes. The gray shading from 0 to 5 ms represents the timespan that was ignored for spike detection, to remove MS artifacts. ***B***, Peristimulus time histograms with raster plots before and after iTBS and (***C***) cTBS delivery. Bin widths were set to 5 ms. ***D***, Changes in spike counts after iTBS/cTBS delivery. Each line corresponds to a single recording point (16 recording points in total for each layer, 4 animals × 4 channels). The changes in baseline spike counts are provided in Extended Data Figure 8-1. ***E***, Box plots of spike count changes. The line plots trace the median spike count changes. Sham (gray), cTBS (blue), and iTBS (orange) groups are shown, with outliers indicated by circles. *p < 0.05, **p < 0.01 (Kruskal–Wallis test followed by post hoc Steel–Dwass test). The box plot for L5 is provided in Extended Data Figure 8-2.

MUA before and after TBS were compared to investigate the modulatory effects of stimulation across different layers of the auditory cortex. Figure 8*A* shows a typical result of spike detection from the high-frequency components of neural activity. Single-MS was delivered at the 0 s mark, where artifacts can be observed. The detected spikes are marked with blue vertical lines.

10.1523/ENEURO.0577-24.2025.f8-1 Figure 8-1 Changes in baseline spike count (the number of spikes occurring 5–50 ms before stimulation) after iTBS/cTBS delivery. Each line corresponds to a single recording point (16 recording points in total for each layer, 4 animals × 4 channels). Download Figure 8-1, TIF file.

10.1523/ENEURO.0577-24.2025.f8-2 Figure 8-2 Box plots of spike count changes in layer 5. The line plots trace the median spike count changes. Sham (gray), continuous theta-burst stimulation (cTBS; blue), and intermittent theta-burst stimulation (iTBS; orange) groups are shown, with outliers indicated by circles. **p < 0.01 (Kruskal–Wallis test followed by *post hoc* Steel–Dwass test). Download Figure 8-2, TIF file.

Figure 8*B* shows typical PSTHs before and after iTBS, and Figure 8*C* displays representative PSTHs before and after cTBS. Changes in spike counts before and after TBS are shown in Figure 8*D*. Compared with the sham condition, the iTBS condition showed minimal differences, whereas the cTBS condition exhibited a moderately lower overall spike count. The baseline spike count stayed relatively stable over time (Extended Data Fig. 8-2).

When the spike counts 5–30 min after TBS were compared with those of the sham group, no significant differences were observed in layers 2/3, 4, or 5 under the iTBS condition (Fig. 8*E*). In contrast, the cTBS condition had significantly lower spike counts in layer 2/3 at 5, 10, and 15 min (5 min, *p* = 0.0014; 10 min, *p* = 0.004; 15 min, *p* = 0.002) and in layer 4 at 5, 10, and 20 min (5 min, *p* = 0.03; 10 min, *p* = 0.001; 20 min, *p* = 0.003; Extended Data Table 4-1, f1 and f2). Additionally, when iTBS and cTBS were compared, there were significant differences in spike counts in layer 2/3 at 5 and 15 min (5 min, *p* = 0.01; 15 min, *p* = 0.03), in layer 4 at 5, 10, and 25 min (5 min, *p* = 0.002; 10 min, *p* = 0.001; 25 min, *p* = 0.03); and in layer 5 (Extended Data Fig. 8-2) at 10 min (*p* = 0.006; Extended Data Table 4-1, f1 and f2).

In summary, iTBS induced minimal changes in spike counts across layers 2/3, 4, and 5 of the auditory cortex, with no significant differences compared with the sham condition. In contrast, cTBS led to significantly lower spike counts in layers 2/3 and 4 at multiple poststimulation time points.

### MUA waveform changes after TBS delivery

**Figure 9.**
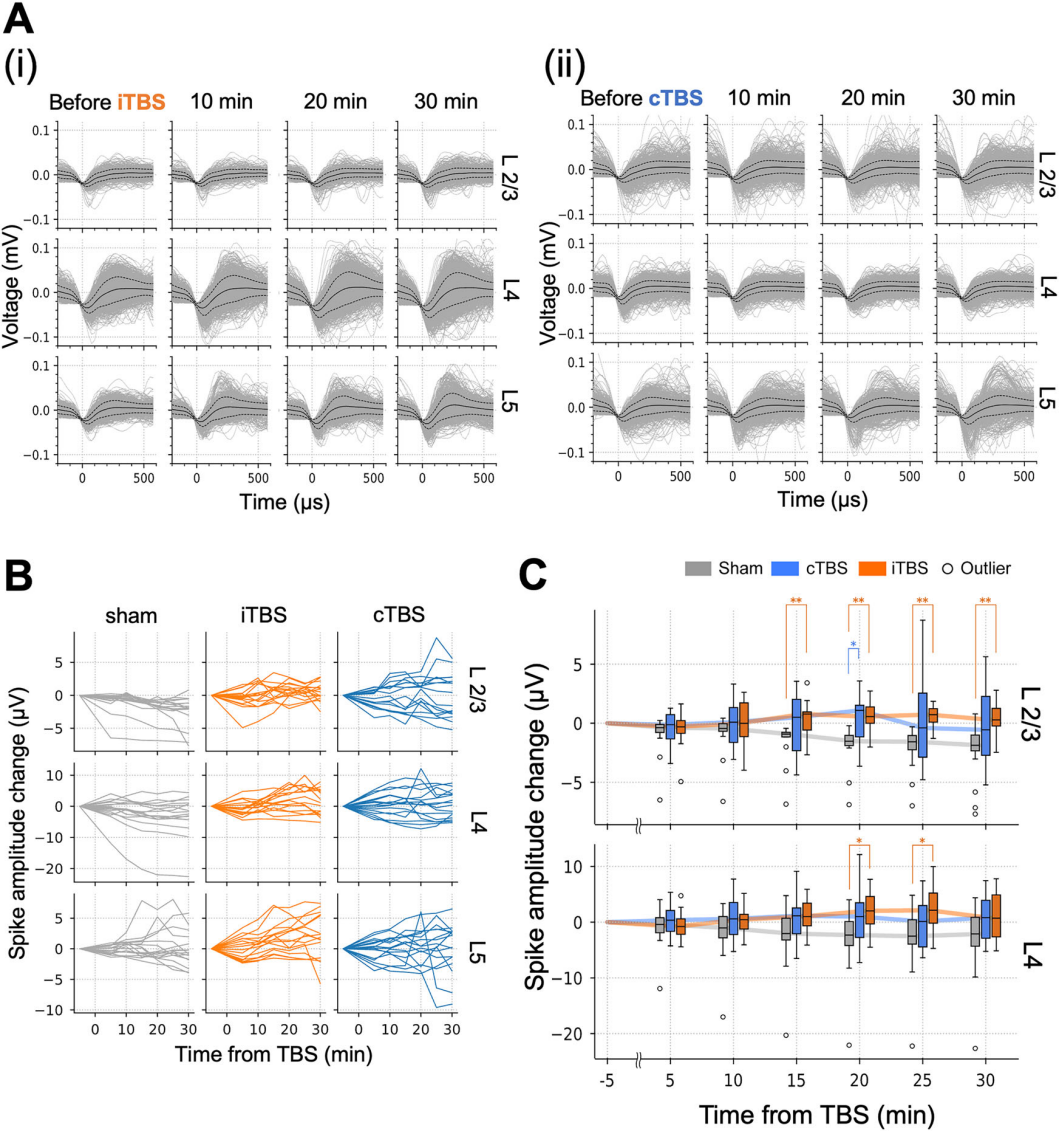
Analysis of multiunit activity (MUA) waveform amplitudes before and after iTBS/cTBS delivery. ***A***, Representative MUA waveforms before and after (***i***) iTBS and (***ii***) cTBS delivery. Recordings from layers 2/3, 4, and 5 (L2/3, L4, and L5, respectively) are shown. The gray lines represent the detected spike waveforms. The black solid lines represent the mean spike waveforms. The black dashed lines represent the standard deviations. ***B***, Changes in spike waveform amplitudes after iTBS/cTBS delivery. Each line corresponds to a single recording point (16 recording points in total for each layer, 4 animals × 4 channels). ***C***, Box plots of spike waveform amplitude changes. The line plots trace the median amplitude changes. Sham (gray), cTBS (blue), and iTBS (orange) groups are shown, with outliers indicated by circles. *p < 0.05, **p < 0.01 (Kruskal–Wallis test followed by post hoc Steel–Dwass test). The box plot for L5 is provided in Extended Data Figure 9-1.

Different neuron types exhibit distinct spike waveforms; however, spike sorting was challenging in the present study because most principal component analysis clusters lacked clear borders. We therefore analyzed the changes in spike amplitudes to understand the excitability of neuron types with large-amplitude spikes. Examples of detected spike waveforms before and after iTBS and cTBS are shown in Figure 9*A*, demonstrating a trend in which the number of high-amplitude spike waveforms gradually increased after the TBS intervention compared with the pre-TBS condition. Figure 9*B* shows the changes in peak amplitudes of spike waveforms 5–30 min after TBS, illustrating an increasing trend in the iTBS condition compared with the sham condition. In contrast, the cTBS condition exhibited considerable variability over time. Statistical analysis revealed significant increases in peak spike amplitude under the iTBS condition, with significant differences observed in layer 2/3 at 15–30 min (15 min, *p* = 0.001; 20 min, *p* = 0.001; 25 min, *p* = 0.001; 30 min, *p* = 0.001) and in layer 4 at 20–25 min (20 min, *p* = 0.014; 25 min, *p* = 0.016; Extended Data Table 4-1, g1 and g2) compared with the sham condition (Fig. 9*C*). For cTBS, a significant difference was observed in layer 2/3 at 20 min (*p* = 0.03; Extended Data Table 4-1, g1 and g2) compared with the sham condition. No significant differences were observed between the iTBS and cTBS conditions.

10.1523/ENEURO.0577-24.2025.f9-1 Figure 9-1 Box plots of spike amplitude changes in layer 5. The line plots trace the median spike count changes. Sham (gray), continuous theta-burst stimulation (cTBS; blue), and intermittent theta-burst stimulation (iTBS; orange) groups are shown, with outliers indicated by circles. Download Figure 9-1, TIF file.

Overall, after iTBS, an increasing trend in high-amplitude spike waveforms was observed, with significant increases in peak spike amplitudes in layers 2/3 and 4 compared with the sham condition. In contrast, cTBS showed more variability, with a significant difference from the sham condition observed only in layer 2/3 at 20 min poststimulation.

## Discussion

In the current study, we constructed a millimeter-sized coil to stimulate localized regions of the mouse brain and then evaluated whether localized TBS can modulate the auditory cortex. First, we measured the physical properties of our coil, which revealed that it was able to induce *B* = 100 mT of magnetic flux density at *V*_pp_ = 40 V. Electric field strength in the auditory cortex at this voltage was estimated by numerical simulation and resulted in *E* = 10 V/m at a depth of 600 µm from the brain surface. Electrophysiological recordings were then made to observe the neural activity of the auditory cortex. Clear LFP responses were visible when tone bursts were presented, confirming that the electrode was indeed inserted in the auditory cortex. Furthermore, single-MS recordings had significantly larger peak amplitudes in living animals than in postmortem animals.

Our analysis of MSEP peak amplitude changes during the TBS sessions revealed no significant differences in iTBS and a significant decrease in cTBS. Further analysis demonstrated a significant decreasing trend during the 2 s iTBS trains. The modulatory effects of TBS were assessed by comparing neural activity among the iTBS, cTBS, and sham conditions. Compared with sham, MSEP peak amplitudes were significantly larger at 5, 10, and 20 min after iTBS. For cTBS, although MSEP peak amplitudes showed a long-lasting decreasing trend, a significant difference compared with sham was only observed at 10 min post-cTBS, likely because of high variability. MUA analysis revealed that only cTBS had an effect on spike counts, which significantly decreased from 5 to 20 min after cTBS delivery. To assess the modulatory effects on spike waveforms, changes in mean spike waveform amplitudes were analyzed, and a significant increase from 15 to 30 min after iTBS was observed. Changes in MUA were observed in layers 2/3 and 4 of the auditory cortex, but not in layer 5.

### MS properties

Because the brain exhibits functional localization, it is crucial to stimulate just a localized region to differentiate its modulatory effect from that of other brain regions. The coil that we constructed had a diameter of 7 mm, which is considerably smaller than the human-specific coils that have been used in other rodent studies (Rotenberg et al., 2010; Vahabzadeh-Hagh et al., 2012). Although we tried using a figure-of-eight coil, we decided not to use it due to difficulties in positioning the silicon probe at the focal point, where the base circuit board of the probe collided with the coil. However, using a thinner probe, such as wire electrodes, could allow compatibility with a figure-of-eight coil.

Our numerical simulation demonstrated that an input voltage of *V*_pp_ = 40 V achieved our numerical target of *E* = 10 V/m at 600 µm from the brain surface. Our numerical target was based on a previous study by Tang et al. (2016b) in which motor cortical activity was modulated using an electric field of *E* = 10 V/m at 700 µm from the brain surface. Because we had a similar electric field intensity, we hypothesized that an input voltage of *V*_pp_ = 40 V would be optimal for modulating layer 4 of the auditory cortex—an important region that receives inputs from the thalamus (Kratz and Manis, 2015)—without stimulating deeper brain regions such as subcortical regions and the brainstem. Notably, iTBS and cTBS had no significant differences in MUA in layer 5 compared with the sham group (Extended Data Fig. 9-1). On the other hand, regions adjacent to the auditory cortex, such as the visual cortex and cerebellum, were likely affected by our stimulation (Fig. 3*D*, Extended Data Fig. 3-1). However, the actual effect on those regions might have been weaker due to the attenuation of the induced electric field by the overlying skin and skull, which were not included in the simulation. In contrast, we believe that the simulated electric field around the recording site closely reflects experimental conditions, as a craniotomy was performed in our in vivo experiment.

The electrical resistance of the coil generates heat, which can modulate neural activity. At *V*_pp_ = 40 V, the temperature increase was 0.4°C with iTBS and 0.7°C with cTBS on the brain surface. Previous research has reported that a temperature increase of >4°C is required to modulate the activity of rat hippocampal neurons (Horváth et al., 2020). It is therefore unlikely that the neural responses obtained from the MS of the prototype coil were caused by heat.

Acoustic measurements showed that the coil generated a maximum SPL of 30.8 dB at 35 kHz. The SPL of the coil is right below the hearing threshold of mice in our laboratory (Extended Data Fig. 2-1*E*–*G*). Thus, given the short duration and low pressure level of the sound, it is unlikely that the mice heard the sound emitted from our coil.

LFP recordings to single-MS showed clear responses with significant differences from the postmortem condition, which confirmed that the observed responses were not MS artifacts. The MS intensity required to induce neural activity remains under debate. A study by Bonmassar et al. (2012) demonstrated that low-intensity MS can induce action potentials in rabbit retinal ganglion cells at an electric field strength of 6 V/m. A more recent study by Osanai et al. (2018) revealed that submillimeter-sized MS with an electric field below 1 V/m induces neural activity in layer 2/3 of the mouse auditory cortex in vivo. However, Tang et al. (2016a) reported that, although rMS with an intensity below 10 V/m increases spike firing rates, it does not directly induce action potentials in layer 5 pyramidal neurons. The thermal and acoustic properties of our coil were appropriate for avoiding neural activity induction; however, additional experiments should be performed to completely rule out the effects of heat and sound, such as by recording single-MS responses from deaf model mice while recording brain surface temperatures. Furthermore, it may be interesting to conduct an experiment delivering sound-TBS, in which only the click sound recorded from the coil is delivered in a TBS pattern; if sound-TBS does not have any modulatory effect, it would support the findings of the present study. In contrast, if sound-TBS has a lasting effect on the auditory cortex, it would indicate that only sound is required to modulate the auditory cortex; this may then become a new noninvasive method for treating hearing disorders.

### Neural activity changes during TBS delivery

Given the limited number of studies that have reported neural activity during TBS application, we investigated MSEP activity changes during TBS delivery. MSEP amplitudes during cTBS showed a slight decreasing trend in MSEP amplitudes over time, consistent with the known inhibitory effects of cTBS on cortical excitability. In contrast, iTBS did not produce significant changes in MSEP amplitudes across the stimulation session. This lack of observable changes over time was unexpected because iTBS is generally considered to produce facilitatory effects. Nonetheless, within the 2 s iTBS trains, we observed a decreasing trend similar to that in the cTBS sessions, in which the first burst consistently elicited a significantly larger MSEP amplitude than that of subsequent bursts. This decrease in neural activity observed in the iTBS trains and cTBS sessions might have been the result of a fatigue-like mechanism, in which neural responses diminish with repeated stimuli, likely reflecting a habituation effect in neural processing (Grill-Spector et al., 2006; Rankin et al., 2009). A previous study by Löfberg et al. (2013) demonstrated that magnetic and auditory repetitive stimulation reduces the amplitude of evoked electroencephalographic potentials, suggesting that neural adaptation occurs not only against sensory responses but also against TMS-evoked activity on a relatively short time scale. Additionally, a recent theta-burst ultrasound stimulation (TBUS) study revealed that intermittent TBUS elicits 20 oscillatory calcium transients synchronized to each train, whereas continuous TBUS induces only one acute calcium transient, at the start of the session (Kim et al., 2024). The calcium transient pattern reported in this previous study resembles the MSEP amplitude changes observed in the present study, and the two findings may share common neurological mechanisms.

### Neural activity changes after TBS delivery

To assess the modulatory effects of TBS on the auditory cortex, we compared changes in neural activity before and after TBS among iTBS, cTBS, and sham conditions. Statistical comparisons revealed a significant increase in MSEP peak amplitudes at 5, 10, and 20 min after iTBS. Studies assessing the modulatory effects of iTBS using MSEPs have reported that these effects lasted for 20 min in humans (Huang et al., 2005) and over 30 min in rodents (Hsieh et al., 2015). Studies evaluating the modulation of iTBS with EEG have reported increased TMS-induced oscillations and lasting facilitatory effects on TMS-evoked potentials in the human cerebellum (Rocchi et al., 2023) and prefrontal cortex (Chung et al., 2017). Additionally, multiple blocks of iTBS increased EEG gamma power and sensory-evoked potentials for several hours in the rodent somatosensory cortex (Benali et al., 2011; Thimm and Funke, 2015). The significant increase in MSEP peak amplitudes in our study aligns with these findings reported in the motor and somatosensory cortices.

We also observed a significant decrease in MSEP amplitude 10 min after cTBS. The modulatory effect of cTBS is thought to last for about an hour in humans (Huang et al., 2005). In a rodent study, a single block of cTBS suppressed MSEP amplitudes for at least 30 min (Hsieh et al., 2015). The inhibitory effect of cTBS on MSEP in our study was relatively weak compared with studies using MSEP measurements. However, this finding is partially consistent with previous rodent studies targeting the somatosensory cortex. For instance, cTBS did not produce long-lasting reductions in EEG power in the somatosensory cortex (Benali et al., 2011), suggesting that its inhibitory effects might be weaker or short-lived in sensory areas. Similarly, a single block of cTBS initially reduced sensory responses in the barrel cortex but later induced a weak facilitatory effect after multiple blocks (Thimm and Funke, 2015).

Another possible explanation for the observed weak inhibitory effects of cTBS on MSEP may be attributed to the frequency of the single-MS used as the evaluation stimulus, which was delivered at 0.2 Hz (5 s intervals). rMS with this frequency is classified as low-frequency rMS, which has inhibitory modulatory effects even at subthreshold intensities (Sebastianelli et al., 2017; Ye et al., 2020). In our findings, MSEP peak amplitudes after the sham TBS condition had a slight reduction (Fig. 7*D*). Thus, the inhibitory effects of single-MS may have diminished the difference between the sham and cTBS conditions. However, despite this inhibitory effect, the observed significant increase in MSEP peak amplitude following iTBS suggests that its actual modulatory effect may be stronger.

To examine the modulatory effects of TBS in auditory cortical layers, we compared the MUA changes before and after TBS. Spike counts before and after TBS revealed no significant differences between iTBS and sham. However, significant reductions in spike counts were observed in layers 2/3 and 4 at 5–20 min after cTBS, which was a longer-lasting modulatory effect than that of the MSEP amplitude change.

To assess changes in spike waveforms, we analyzed changes in spike waveform amplitudes with MUAs because it was challenging to sort the spike waveforms into single units. Interestingly, both iTBS and cTBS had a slight increasing trend, whereas the sham condition showed a decreasing trend. A significant increase in amplitudes was observed primarily for iTBS in layer 2/3; this increase started 15 min after iTBS delivery. The increase in mean spike amplitudes might have resulted from increased amplitudes of each spike or from increased numbers of high-amplitude spikes. In extracellular recordings, changes in spike amplitudes are most often caused by changes in the positions of electrodes relative to neurons. Although there is a high chance that the electrode placement changed over time, it seems unlikely that positional changes would have occurred in the same manner for all animals in each condition, leading to a significant difference. We therefore hypothesize that the number of active neuron types—namely, fast-spiking neurons and regular-spiking neurons—changed in our study. Regular-spiking neurons, such as pyramidal neurons, are thought to have a wider and larger waveform than fast-spiking neurons, such as parvalbumin-positive interneurons, which typically have narrow waveforms (Chen et al., 1996). A previous study reported that parvalbumin expression decreases 20–40 min after iTBS delivery (Hoppenrath and Funke, 2013). Parvalbumin-positive neurons have higher firing rates than pyramidal neurons (Middleton et al., 2012) and inhibit 20–25% of neighboring pyramidal neurons (Thomson et al., 1996), thereby controlling the spike timing of pyramidal neurons (Moore and Wehr, 2013; Hijazi et al., 2023). Our observed increase in mean spike amplitudes without a change in overall spike counts following iTBS may therefore be explained by decreased parvalbumin-positive neuron activity coupled with increased pyramidal cell activity. This shift in active cell types might result in larger average spike amplitudes while maintaining consistent overall spike counts, because the reduction in high-frequency spiking from parvalbumin-positive neurons may be counterbalanced by the increased activity of disinhibited pyramidal neurons. While we observed changes in spike waveform amplitude following iTBS and cTBS, we acknowledge that interpreting these changes in detail is challenging due to the nature of MUA recordings. Changes in spike waveform amplitude may indicate changes in neuronal activity or shifts in the proportion of activated neuron types (e.g., changes in the ratio of excitatory regular-spiking to inhibitory fast-spiking neurons). However, MUA does not allow precise waveform characterization, making it difficult to identify individual neuron types or their specific contributions. Further investigations using single-unit recordings or intracellular measurements are necessary to clarify mechanisms at the single-cell level.

Combining the results of our MSEP and MUA analyses, we observed an increase in MSEP activity 5–20 min after iTBS, which was followed by an increase in MUA activity after 15 min. It has previously been reported that the expression levels of immediate early genes and glutamate decarboxylase 2 (all markers of neuronal plasticity) increase 10–20 min after iTBS, whereas the expression levels of calcium-binding proteins (parvalbumin and calbindin) and glutamate decarboxylase 1 decrease 20–40 min after iTBS (Hoppenrath and Funke, 2013). The timing of these changes aligns with the timing of MSEP and MUA changes observed after iTBS in our study, thus implying an association between gene expression changes and electrophysiological activity changes.

### Limitations and future directions

One limitation of our study is the use of a multielectrode array during rTMS, which may introduce the possibility of microstimulation. However, while the induced electric field from magnetic stimulation may generate small, induced currents in the electrodes, these currents are expected to be minimal. A previous study has described a method for measuring the amount of TMS-induced charge injection and showed that, although transcranial magnetic stimulation can produce a small current, it remains below the threshold required to evoke neuronal activity in intracortical microstimulation (Li et al., 2017). Due to the lack of detailed circuit specifications from the amplifier manufacturer—particularly the input capacitance (*C*_in_)—we could not precisely calculate the charge injection. However, using an assumed capacitance (*C*_in_ = 30 pF) and the measured voltage change of the MS artifact (dV/dt = 110.4 V/s), we estimated the charge injection to be approximately 3.31 nA; i.e., $I(t)=C_{\mathrm{in}}\cdot \mathrm{dV}/\mathrm{dt}=30\times 10^{-12}\;[F]\times 110.4\;[V/s]=3.31\;[\mathrm{nA}]$. The current required to induce microstimulation is on the order of microamperes (Voigt et al., 2017; Allison-Walker et al., 2021). Given that our estimated current is orders of magnitude lower, it is unlikely to cause microstimulation. Therefore, the primary factor influencing brain activity in our setup is the direct effect of magnetic stimulation, rather than unintended microstimulation from induced currents in the recording electrodes.

In the present study, we focused on the modulatory effects of TBS on neurons; however, it is worth noting that non-neuronal cell types, such as glial cells, can also be affected by rTMS. Astrocytes, which provide nutrients to neurons and contribute to the formation of the blood–brain barrier, experience a reduction in the expression of genes specifically related to inflammation and calcium signaling following 1 and 10 Hz low-intensity rMS (Clarke et al., 2021). Additionally, 14 d of iTBS promotes the survival and maturation of oligodendrocytes (Cullen et al., 2019) and expands gaps in the myelin sheath (nodes of Ranvier), thus reducing the propagation speed of membrane potentials (Cullen et al., 2021). Furthermore, not only glial cells but also cerebral blood flow can be influenced by rTMS. Blood flow is an indirect biomarker of neuronal activity, with neurons and astrocytes acting on arterial smooth muscle in response to glutamate release (Attwell et al., 2010). Moreover, one study reported that electromagnetic stimulation causes smooth muscle contraction (Blanquer et al., 2022), suggesting that MS might directly increase blood flow. An investigation of the modulatory effects of TBS on non-neuronal factors (such as glial cells and blood flow) in addition to neurons will provide a more detailed understanding of the mechanisms of TBS.

rTMS has been explored as a potential treatment for tinnitus, which is a condition in which a phantom sound is perceived without any external auditory stimulus. The evidence level of rTMS as a treatment for tinnitus is considered low (Lefaucheur et al., 2020) because two large clinical trials have reported conflicting results: Folmer et al. (2015) demonstrated that 1 Hz rTMS reduced tinnitus in 64 patients, whereas Landgrebe et al. (2017) revealed no significant effects in a larger sample of 146 patients. However, several studies have noted that tinnitus is associated with hyperactivity in auditory brain regions (Noreña and Eggermont, 2003; Mulders and Robertson, 2009). Thus, although the precise mechanism of tinnitus suppression by rTMS is not yet fully understood, it has been hypothesized that reducing this hyperactivity may contribute to the suppression of tinnitus (Zhang, 2013). To our knowledge, the present study is the first to assess the effects of TBS in the auditory cortex of mice. We measured changes in neural activity to single-MS instead of sound, with the aim of evaluating the modulatory effects at the point at which TBS was applied. As a next step, the modulatory effects on sound responses and tinnitus activity should be studied, to determine whether our low-intensity TBS protocol is suitable for tinnitus suppression in mice. Additionally, conducting repetitive auditory stimulation in an iTBS or cTBS pattern could be interesting, as it may serve as a potential noninvasive treatment for hearing disorders if it induces a modulation effect. Conversely, if no modulation effect is observed, it could function as a sham condition for magnetic TBS experiments. Furthermore, the use of recently developed tools such as optogenetics might be useful for understanding the circuit-level mechanisms of rTMS-induced tinnitus suppression.

## Conclusion

In the present study, we used a millimeter-sized coil to demonstrate that localized TBS was able to induce bidirectional modulation of the mouse auditory cortex. Electrophysiological measurements revealed that repetition suppression occurred during TBS delivery; decreasing trends in MSEP amplitudes were observed within a train in iTBS and within a whole session in cTBS. By analyzing the changes in neural activity before and after TBS, a bidirectional modulatory effect was observed in which MSEP amplitudes increased for 20 min after iTBS and decreased for 10 min after cTBS. Although the inhibitory effects of cTBS were weak in terms of MSEPs, a significant decrease in spike counts was observed 20 min after cTBS. Additionally, iTBS had a delayed facilitatory effect on spike amplitudes, which matches the reported time course of decreased parvalbumin expression from previous research. Given the limited number of studies assessing the modulatory effects of TBS on the auditory cortex of rodents, our findings provide valuable physiological evidence of TBS modulation in the mouse auditory cortex. Nonetheless, further research is needed to fully understand the mechanisms underlying how rTMS modulates the auditory system and to be able to treat certain hearing disorders, such as tinnitus.

## Synthesis

Reviewing Editor: Christine Portfors, Washington State University Decisions are customarily a result of the Reviewing Editor and the peer reviewers coming together and discussing their recommendations until a consensus is reached. When revisions are invited, a fact-based synthesis statement explaining their decision and outlining what is needed to prepare a revision will be listed below. The following reviewer(s) agreed to reveal their identity: NONE.

The reviewers found many strengths with the study and believe it to be an important addition to the literature. Several points need to be addressed however to clarify aspects of the manuscript to make the results more clear and strengthen the interpretation.

Specific comments:

Reviewer 1:

- Please specify what type of anesthetics the authors used throughout the experiments, especially during electrophysiological recording.

- Although the SPL generated by the coil was ~30 dB, which is below the hearing threshold of mice in the authors' laboratory, it would be more convincing that the coil-generated sound does not affect neural responses if the authors could demonstrate the absence of cortical responses to a similar sound level, such as 30 dB white noise.

- In Fig. 5B, it looks there are positive amplitude response immediately after MS in iTBS but they are not observed in cTBS. Is this robust phenomenon across mice? If so, can the authors speculate where the difference comes from?

- Although the authors investigated how LFP amplitudes change within each iTBS train, it would be helpful if the authors also examine the changes within each cTBS train. This would allow readers to compare how the peak amplitude drops differently or similarly across trains between iTBS and cTBS.

- Although the authors analyzed LFP responses to MS before and after iTBS/ cTBS in Fig. 7, did the authors investigate neural responses to auditory stimulation and spontaneous activities before/after TBS? This would further support the changes in cortical excitability induced by TBS.

- Regarding the authors' findings in lines 342-358, it would be helpful for readers if the authors summarize clearly the extent to which the findings on the modulatory effects of iTBS and cTBS are consistent or inconsistent with previous reports with stimulations in the motor cortex, or whether these findings are novel.

- Can the authors clarify what interpretations can be inferred about the function of neurons and the brain based on changes in spike waveforms?

Minor comments:

- In Introduction line 36-39, "Theta-burst stimulation (TBS), first reported by Huang et al. (2005), is a complex stimulation pattern in which triplet pulses with a 20 ms inter-pulse interval (50 Hz), called "bursts," are presented at an inter-burst interval of 200 ms (5 Hz).":

This is a little confusing because theta-burst stimulation itself has been used in electric stimulation since Larson and Lynch, Science (1986). It would be clearer to specify magnetic stimulation in this sentense.

- In line 151-152, "The sound waveform without the MS artifact was obtained by subtracting the baseline waveform from the waveform recorded without the acrylic board.", can you show the speculated sound waveform in Extended Figure 2-1?

- Can the authors specify which cortical layer the recorded LFP waveforms shown in Fig. 4-7 were obtained from?

- In Fig. 5B and 6B, do the circles indicate LFP peak amplitude? Please specify these.

Reviewer 2:

1. The authors used a simplified brain model for E-field simulation. Please clarify the impact of excluding components such as the skull, skin, cerebrospinal fluid, and other tissues on the accuracy of the simulation results.

2. Did the author calibrate the Hall effect sensor using a known standard?

3. Was MS/TBS performed while the animal was under general anesthesia? If so, what anesthetics were administered? Please clarify the impact of the anesthetics on the stimulation effects.

4. Frequencies of 4, 8, and 16 kHz were used as the stimulus sound for each FAI trial. Which frequency was used to determine the insertion point for the electrode? How was consistency ensured across different animals?

5. Please clarify the influence of the multi-electrode array and the metal rod on the induced electric field. Could the electrodes or metal components cause micro-stimulation to the brain?

6. Did the coil stimulate only the auditory cortex? If not, please clarify the influence of stimulating a broader brain region.

7. Please clarify the rationale for choosing a circular coil instead of a more focal coil, such as a figure-of-eight coil.

8. It is confusing to use both 50 Hz and 300 Hz low-pass filters to analyze LFPs in different cases. Please provide further clarification on which scenarios require each low-pass filter.

9. It would be more accurate to use the term "MS-evoked potentials" instead of "LFPs" to describe the time-locked signals elicited by magnetic stimulation.

10. What is the rationale for using the negative peak amplitude of LFPs for analysis?

11. The authors only measured the temperature change of the coil. Since the coil was placed very close to the brain surface, what was the temperature change of the brain surface?

12. The authors claimed that single-MS had minimal acoustic effects. It would be better to validate this by using the same acoustic pattern without magnetic stimulation to determine whether there is a neural response. In addition, what would be the results if the acoustic effects of iTBS and cTBS were maintained during sham stimulation?

13. Please provide more details on how the authors compared the LFP responses between sound and single-MS. How many trials and animals were used in the analysis? The gray traces in Figure 4B appear very noisy. Are these raw data, and what is the reason for the inconsistency?

14. How did the authors determine the LEP peak amplitude in response to iTBS and cTBS (Fig 5 &6)? The peak amplitude of the average traces may not accurately reflect the true peak amplitude for individual traces. Did all peak values have similar latency?

15. Was there a baseline drift in spiking activities over time? If so, what could be the underlying cause? (Fig 8 &9)

## References

[B1] Allen Institute for Brain Science (2004) Allen mouse brain atlas.

[B2] Allison-Walker T, Hagan MA, Price NSC, Wong YT (2021) Microstimulation-evoked neural responses in visual cortex are depth dependent. Brain Stimul 14:741–750. 10.1016/j.brs.2021.04.020 33975054

[B3] Attwell D, Buchan AM, Charpak S, Lauritzen M, MacVicar BA, Newman EA (2010) Glial and neuronal control of brain blood flow. Nature 468:232–243. 10.1038/nature09613 21068832 PMC3206737

[B4] Bai Z, Zhang J, Fong KNK (2022) Effects of transcranial magnetic stimulation in modulating cortical excitability in patients with stroke: a systematic review and meta-analysis. J Neuroeng Rehabil 19:24. 10.1186/s12984-022-00999-4 35193624 PMC8862292

[B5] Bakker R, Tiesinga P, Kötter R (2015) The scalable brain atlas: instant web-based access to public brain atlases and related content. Neuroinformatics 13:353–366. 10.1007/s12021-014-9258-x 25682754 PMC4469098

[B6] Benali A, Trippe J, Weiler E, Mix A, Petrasch-Parwez E, Girzalsky W, Eysel UT, Erdmann R, Funke K (2011) Theta-burst transcranial magnetic stimulation alters cortical inhibition. J Neurosci 31:1193–1203. 10.1523/JNEUROSCI.1379-10.2011 21273404 PMC6623597

[B7] Blanquer A, Careta O, Anido-Varela L, Aranda A, Ibáñez E, Esteve J, Nogués C, Murillo G (2022) Biocompatibility and electrical stimulation of skeletal and smooth muscle cells cultured on piezoelectric nanogenerators. Int J Mol Sci 23:432. 10.3390/ijms23010432 35008860 PMC8745485

[B8] Bonmassar G, Lee SW, Freeman DK, Polasek M, Fried SI, Gale JT (2012) Microscopic magnetic stimulation of neural tissue. Nat Commun 3:921. 10.1038/ncomms1914 22735449 PMC3621430

[B9] Che X, Cash RFH, Luo X, Luo H, Lu X, Xu F, Zang Y-F, Fitzgerald PB, Fitzgibbon BM (2021) High-frequency rTMS over the dorsolateral prefrontal cortex on chronic and provoked pain: a systematic review and meta-analysis. Brain Stimul 14:1135–1146. 10.1016/j.brs.2021.07.004 34280583

[B10] Chen W, Zhang J-J, Hu G-Y, Wu C-P (1996) Electrophysiological and morphological properties of pyramidal and nonpyramidal neurons in the cat motor cortex in vitro. Neuroscience 73:39–55. 10.1016/0306-4522(96)00009-7 8783228

[B11] Chervyakov AV, Chernyavsky AY, Sinitsyn DO, Piradov MA (2015) Possible mechanisms underlying the therapeutic effects of transcranial magnetic stimulation. Front Hum Neurosci 9:303. 10.3389/fnhum.2015.00303 26136672 PMC4468834

[B12] Chung SW, Lewis BP, Rogasch NC, Saeki T, Thomson RH, Hoy KE, Bailey NW, Fitzgerald PB (2017) Demonstration of short-term plasticity in the dorsolateral prefrontal cortex with theta burst stimulation: a TMS-EEG study. Clin Neurophysiol 128:1117–1126. 10.1016/j.clinph.2017.04.005 28511124

[B13] Clarke D, Beros J, Bates KA, Harvey AR, Tang AD, Rodger J (2021) Low intensity repetitive magnetic stimulation reduces expression of genes related to inflammation and calcium signalling in cultured mouse cortical astrocytes. Brain Stimul 14:183–191. 10.1016/j.brs.2020.12.007 33359601

[B14] Cullen CL, et al. (2021) Periaxonal and nodal plasticities modulate action potential conduction in the adult mouse brain. Cell Rep 34:108641. 10.1016/j.celrep.2020.108641 33472075

[B15] Cullen CL, Senesi M, Tang AD, Clutterbuck MT, Auderset L, O’Rourke ME, Rodger J, Young KM (2019) Low-intensity transcranial magnetic stimulation promotes the survival and maturation of newborn oligodendrocytes in the adult mouse brain. Glia 67:1462–1477. 10.1002/glia.23620 30989733 PMC6790715

[B16] Folmer RL, Theodoroff SM, Casiana L, Shi Y, Griest S, Vachhani J (2015) Repetitive transcranial magnetic stimulation treatment for chronic tinnitus: a randomized clinical trial. JAMA Otolaryngol Head Neck Surg 141:716–722. 10.1001/jamaoto.2015.1219 26181507

[B17] Grill-Spector K, Henson R, Martin A (2006) Repetition and the brain: neural models of stimulus-specific effects. Trends Cogn Sci 10:14–23. 10.1016/j.tics.2005.11.006 16321563

[B18] Herreras O (2016) Local field potentials: myths and misunderstandings. Front Neural Circuits 10:101. 10.3389/fncir.2016.00101 28018180 PMC5156830

[B19] Hijazi S, Smit AB, van Kesteren RE (2023) Fast-spiking parvalbumin-positive interneurons in brain physiology and Alzheimer’s disease. Mol Psychiatry 28:4954–4967. 10.1038/s41380-023-02168-y 37419975 PMC11041664

[B20] Hoppenrath K, Funke K (2013) Time-course of changes in neuronal activity markers following iTBS-TMS of the rat neocortex. Neurosci Lett 536:19–23. 10.1016/j.neulet.2013.01.003 23328445

[B21] Horváth ÁC, Borbély S, Boros ÖC, Komáromi L, Koppa P, Barthó P, Fekete Z (2020) Infrared neural stimulation and inhibition using an implantable silicon photonic microdevice. Microsyst Nanoeng 6:1–12. 10.1038/s41378-020-0153-3 34567656 PMC8433474

[B22] Hsieh T-H, Huang Y-Z, Rotenberg A, Pascual-Leone A, Chiang Y-H, Wang J-Y, Chen J-JJ (2015) Functional dopaminergic neurons in substantia nigra are required for transcranial magnetic stimulation-induced motor plasticity. Cereb Cortex 25:1806–1814. 10.1093/cercor/bht421 24451657

[B23] Huang Y-Z, Edwards MJ, Rounis E, Bhatia KP, Rothwell JC (2005) Theta burst stimulation of the human motor cortex. Neuron 45:201–206. 10.1016/j.neuron.2004.12.033 15664172

[B24] Jannati A, Oberman LM, Rotenberg A, Pascual-Leone A (2023) Assessing the mechanisms of brain plasticity by transcranial magnetic stimulation. Neuropsychopharmacology 48:191–208. 10.1038/s41386-022-01453-8 36198876 PMC9700722

[B25] Kim H-J, et al. (2024) Long-lasting forms of plasticity through patterned ultrasound-induced brainwave entrainment. Sci Adv 10:eadk3198. 10.1126/sciadv.adk3198 38394205 PMC10889366

[B26] Klomjai W, Katz R, Lackmy-Vallée A (2015) Basic principles of transcranial magnetic stimulation (TMS) and repetitive TMS (rTMS). Ann Phys Rehabil Med 58:208–213. 10.1016/j.rehab.2015.05.005 26319963

[B27] Kratz MB, Manis PB (2015) Spatial organization of excitatory synaptic inputs to layer 4 neurons in mouse primary auditory cortex. Front Neural Circuits 9:17. 10.3389/fncir.2015.00017 25972787 PMC4413692

[B28] Landgrebe M, et al. (2017) 1-Hz rTMS in the treatment of tinnitus: a sham-controlled, randomized multicenter trial. Brain Stimul 10:1112–1120. 10.1016/j.brs.2017.08.001 28807845

[B29] Lefaucheur J-P, et al. (2020) Evidence-based guidelines on the therapeutic use of repetitive transcranial magnetic stimulation (rTMS): an update (2014–2018). Clin Neurophysiol 131:474–528. 10.1016/j.clinph.2019.11.002 31901449

[B30] Li B, Virtanen JP, Oeltermann A, Schwarz C, Giese MA, Ziemann U, Benali A (2017) Lifting the veil on the dynamics of neuronal activities evoked by transcranial magnetic stimulation. Elife 6:e30552. 10.7554/eLife.30552 29165241 PMC5722613

[B31] Löfberg O, Julkunen P, Tiihonen P, Pääkkönen A, Karhu J (2013) Repetition suppression in the cortical motor and auditory systems resemble each other – a combined TMS and evoked potential study. Neuroscience 243:40–45. 10.1016/j.neuroscience.2013.03.060 23570793

[B32] Middleton JW, Omar C, Doiron B, Simons DJ (2012) Neural correlation is stimulus modulated by feedforward inhibitory circuitry. J Neurosci 32:506–518. 10.1523/JNEUROSCI.3474-11.2012 22238086 PMC3282531

[B33] Moore AK, Wehr M (2013) Parvalbumin-expressing inhibitory interneurons in auditory cortex are well-tuned for frequency. J Neurosci 33:13713–13723. 10.1523/JNEUROSCI.0663-13.2013 23966693 PMC3755717

[B34] Mulders WHAM, Leggett K, Mendis V, Tarawneh H, Wong JK, Rodger J (2019) Low-intensity repetitive transcranial magnetic stimulation over prefrontal cortex in an animal model alters activity in the auditory thalamus but does not affect behavioural measures of tinnitus. Exp Brain Res 237:883–896. 10.1007/s00221-018-05468-w 30649586

[B35] Mulders WH, Robertson D (2009) Hyperactivity in the auditory midbrain after acoustic trauma: dependence on cochlear activity. Neuroscience 164:733–746. 10.1016/j.neuroscience.2009.08.036 19699277

[B36] Mulders WHAM, Vooys V, Makowiecki K, Tang AD, Rodger J (2016) The effects of repetitive transcranial magnetic stimulation in an animal model of tinnitus. Sci Rep 6:38234. 10.1038/srep38234 27905540 PMC5131273

[B37] Noreña AJ, Eggermont JJ (2003) Changes in spontaneous neural activity immediately after an acoustic trauma: implications for neural correlates of tinnitus. Hear Res 183:137–153. 10.1016/S0378-5955(03)00225-9 13679145

[B38] Osanai H, Minusa S, Tateno T (2018) Micro-coil-induced inhomogeneous electric field produces sound-driven-like neural responses in microcircuits of the mouse auditory cortex in vivo. Neuroscience 371:346–370. 10.1016/j.neuroscience.2017.12.008 29246784

[B39] Rankin CH, et al. (2009) Habituation revisited: an updated and revised description of the behavioral characteristics of habituation. Neurobiol Learn Mem 92:135–138. 10.1016/j.nlm.2008.09.012 18854219 PMC2754195

[B40] Rocchi L, Spampinato D, Pezzopane V, Orth M, Bisiacchi P, Rothwell J, Casula E (2023) Cerebellar noninvasive neuromodulation influences the reactivity of the contralateral primary motor cortex and surrounding areas: a TMS-EMG-EEG study. Cerebellum 22:319–331. 10.1007/s12311-022-01398-0 35355218

[B41] Rotenberg A, Muller PA, Vahabzadeh-Hagh AM, Navarro X, López-Vales R, Pascual-Leone A, Jensen F (2010) Lateralization of forelimb motor evoked potentials by transcranial magnetic stimulation in rats. Clin Neurophysiol 121:104–108. 10.1016/j.clinph.2009.09.008 19900839 PMC2818443

[B42] Scheper A, Rosenfeld C, Dubljević V (2022) The public impact of academic and print media portrayals of TMS: shining a spotlight on discrepancies in the literature. BMC Med Ethics 23:25. 10.1186/s12910-022-00760-5 35282833 PMC8919547

[B43] Sebastianelli L, Versace V, Martignago S, Brigo F, Trinka E, Saltuari L, Nardone R (2017) Low-frequency rTMS of the unaffected hemisphere in stroke patients: a systematic review. Acta Neurol Scand 136:585–605. 10.1111/ane.12773 28464421

[B44] Suppa A, Huang Y-Z, Funke K, Ridding MC, Cheeran B, Di Lazzaro V, Ziemann U, Rothwell JC (2016) Ten years of theta burst stimulation in humans: established knowledge, unknowns and prospects. Brain Stimul 9:323–335. 10.1016/j.brs.2016.01.006 26947241

[B46] Tang AD, Hong I, Boddington LJ, Garrett AR, Etherington S, Reynolds JNJ, Rodger J (2016a) Low-intensity repetitive magnetic stimulation lowers action potential threshold and increases spike firing in layer 5 pyramidal neurons in vitro. Neuroscience 335:64–71. 10.1016/j.neuroscience.2016.08.030 27568058

[B45] Tang AD, et al. (2016b) Construction and evaluation of rodent-specific rTMS coils. Front Neural Circuits 10:47. 10.3389/fncir.2016.00047 27445702 PMC4928644

[B47] Thimm A, Funke K (2015) Multiple blocks of intermittent and continuous theta-burst stimulation applied via transcranial magnetic stimulation differently affect sensory responses in rat barrel cortex. J Physiol 593:967–985. 10.1113/jphysiol.2014.282467 25504571 PMC4398532

[B48] Thomson AM, West DC, Hahn J, Deuchars J (1996) Single axon IPSPs elicited in pyramidal cells by three classes of interneurones in slices of rat neocortex. J Physiol 496:81–102. 10.1113/jphysiol.1996.sp021667 8910198 PMC1160826

[B49] Vahabzadeh-Hagh AM, Muller PA, Gersner R, Zangen A, Rotenberg A (2012) Translational neuromodulation: approximating human transcranial magnetic stimulation protocols in rats. Neuromodulation 15:296–305. 10.1111/j.1525-1403.2012.00482.x 22780329 PMC5764706

[B50] Voigt MB, Hubka P, Kral A (2017) Intracortical microstimulation differentially activates cortical layers based on stimulation depth. Brain Stimul 10:684–694. 10.1016/j.brs.2017.02.009 28284918

[B51] Ye E, Lee S, Park W, Park E, Cho D-W, Jang J, Park S-M (2020) In vitro study of neurochemical changes following low-intensity magnetic stimulation. IEEE Access 8:194363–194372. 10.1109/ACCESS.2020.3033029

[B52] Zhang J (2013) Auditory cortex stimulation to suppress tinnitus: mechanisms and strategies. Hear Res 295:38–57. 10.1016/j.heares.2012.05.007 22683861

[B53] Zimdahl JW, Thomas H, Bolland SJ, Leggett K, Barry KM, Rodger J, Mulders WHAM (2021) Excitatory repetitive transcranial magnetic stimulation over prefrontal cortex in a Guinea pig model ameliorates tinnitus. Front Neurosci 15:693935. 10.3389/fnins.2021.693935 34366777 PMC8339289

