# Supplementary figures and images for: Localized Theta-Burst Magnetic Stimulation Induces Bidirectional Neural Modulation in the Mouse Auditory Cortex In Vivo
Source: eNeuro. 2025 May 2;12(5):ENEURO.0577-24.2025. doi: 10.1523/ENEURO.0577-24.2025 (PMC12077811; doi:10.1523/ENEURO.0577-24.2025)

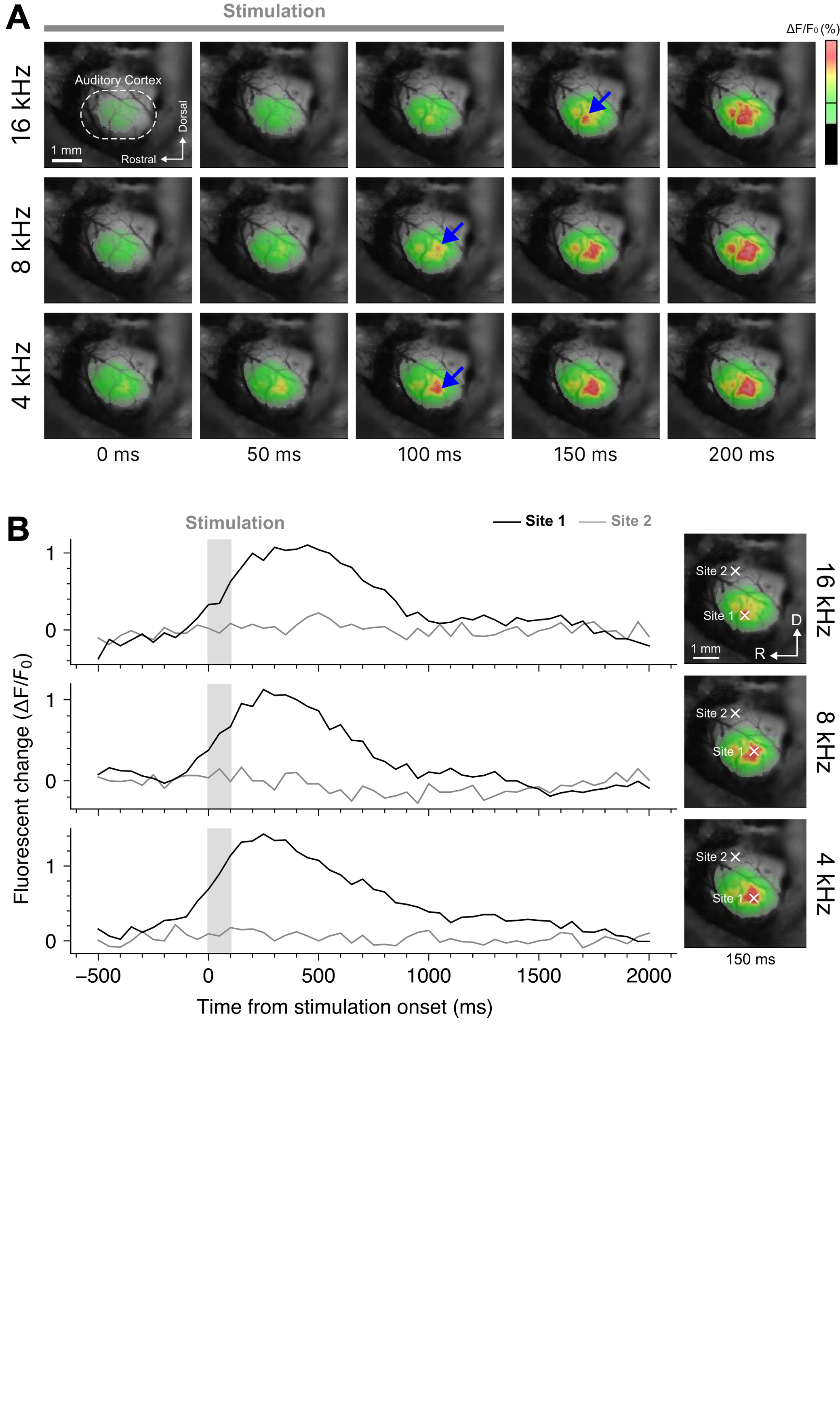

Supplement: Figure 1-1 — Verification of auditory cortex targeting by flavoprotein autofluorescence imaging. A, Auditory cortical activity in response to 4, 8, and 16 kHz tone-burst exposure. Blue arrows indicate the starting point of the response. B, Line plots of fluorescent intensity at the starting point of the response (site 1, black line) and a nearby point outside the auditory cortex (site 2, gray line). Download Figure 1-1, TIF file. [file eneuro-12-ENEURO.0577-24.2025-s003.tif]

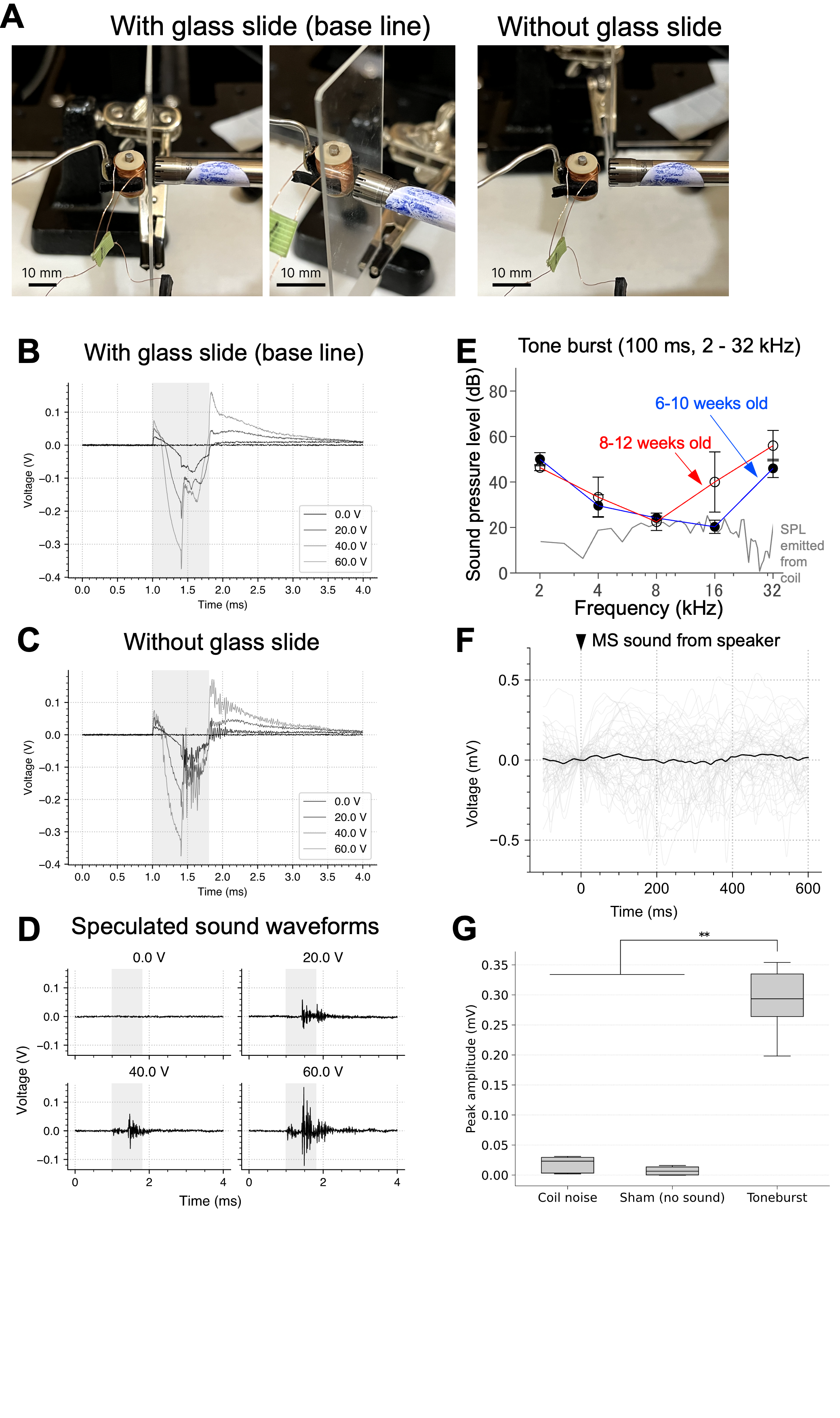

Supplement: Figure 2-1 — Sound emission measurements from the coil. A, Placement of the coil and microphone with and without the glass slide. B, Average waveform of the recorded signal with a microscope glass slide inserted between the coil and the microphone, C, without the glass slide, and D, the speculated sound waveform obtained by subtracting the waveform recorded with the glass slide from that without the glass slide. The result for 40 V was previously shown in Fig. 2D. E, Hearing thresholds of the mice in our lab (n = 5, 6–10 weeks old). The hearing threshold was defined as the lowest tone-burst (100 ms, 2–32 kHz) intensity evoking an auditory brainstem response (ABR). The average ABR waveform of 512 trials was used for the hearing threshold detection. Initial hearing thresholds are shown in blue. Hearing thresholds recorded 2 weeks after the initial recording (8–12 weeks old) are shown in red. Error bars indicate the standard error. The gray line indicates the sound pressure level of the sound emitted from our coil. F, Representative LFP waveforms in response to the MS sound (Fig. 2D) played from the speaker. The MS sound was calibrated to achieve a maximum sound pressure level of 30.8 dB in its frequency components, ensuring consistency with Fig. 2E. Plot formats are the same as in Fig. 4. G. Comparison of the peak amplitude across coil noise, sham (no sound), and tone-burst conditions (9 recording points per condition, 3 animals × 3 channels). **p < 0.01 (Kruskal–Wallis test followed by post hoc Steel–Dwass test) h1, h2. Download Figure 2-1, TIF file. [file eneuro-12-ENEURO.0577-24.2025-s002.tif]

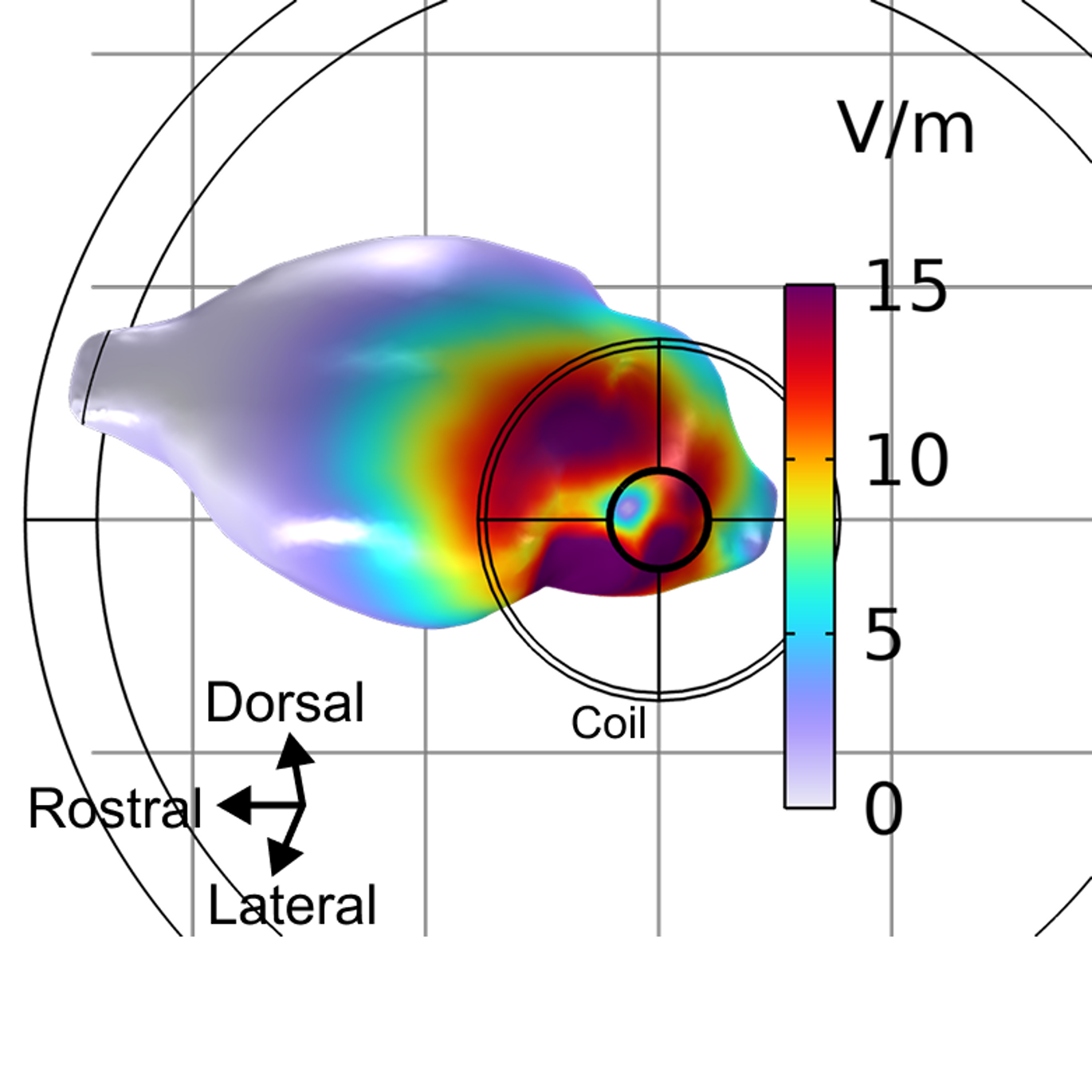

Supplement: Figure 3-1 — Top-down view of Fig. 3D, showing the spatial distribution of the simulated electric field (V/m) induced by the coil. The color scale represents the electric field strength, with warmer colors indicating higher intensities. Download Figure 3-1, TIF file. [file eneuro-12-ENEURO.0577-24.2025-s004.tif]

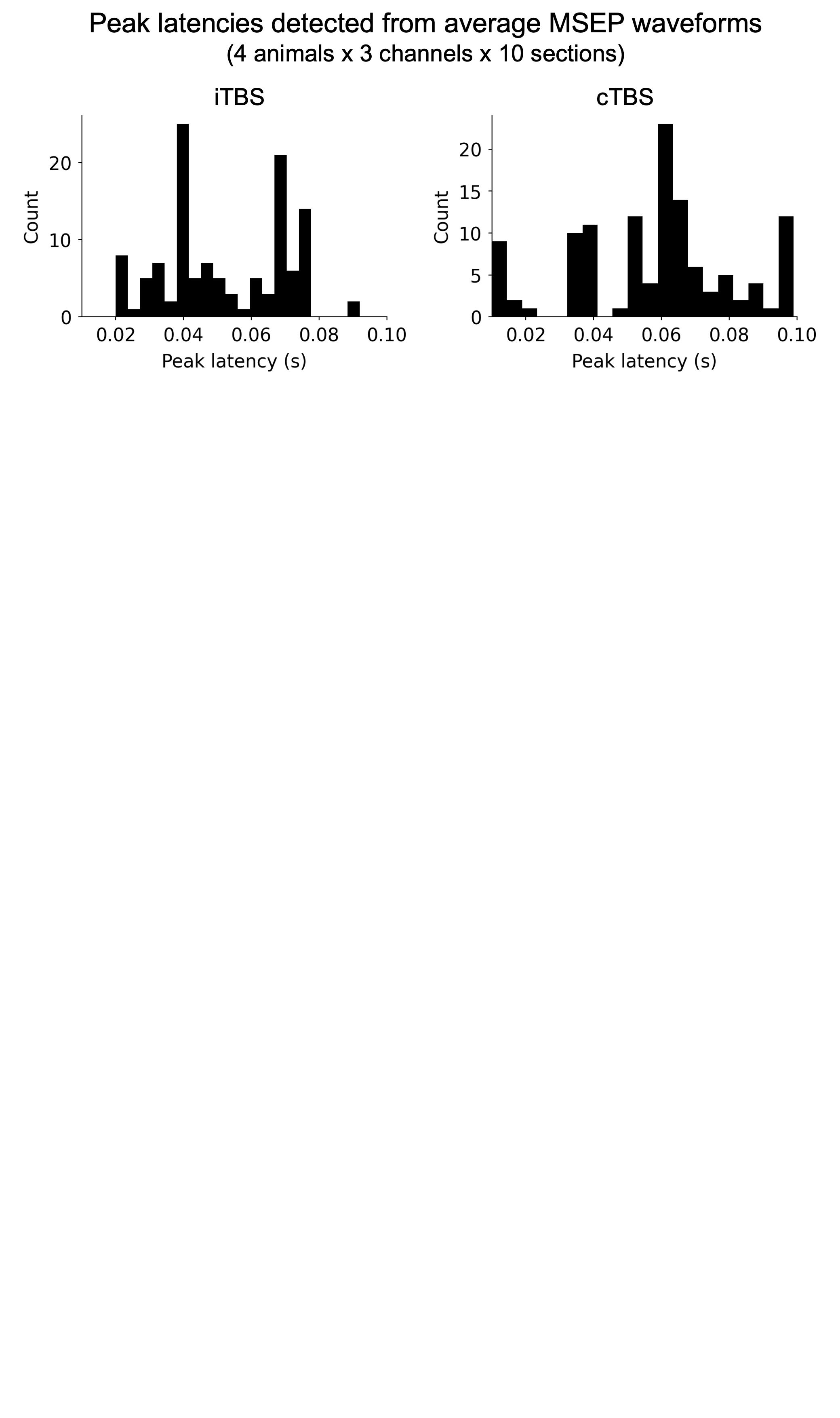

Supplement: Figure 5-1 — Histograms of peak latencies detected from average MSEP waveforms during iTBS and cTBS. Data from all sections in iTBS/cTBS (see Fig. 5 for details) were included (10 sections × 4 animals × 3 channels). The peaks had similar latencies, around 40 and 70 ms for iTBS and 60 ms for cTBS. Download Figure 5-1, TIF file. [file eneuro-12-ENEURO.0577-24.2025-s005.tif]

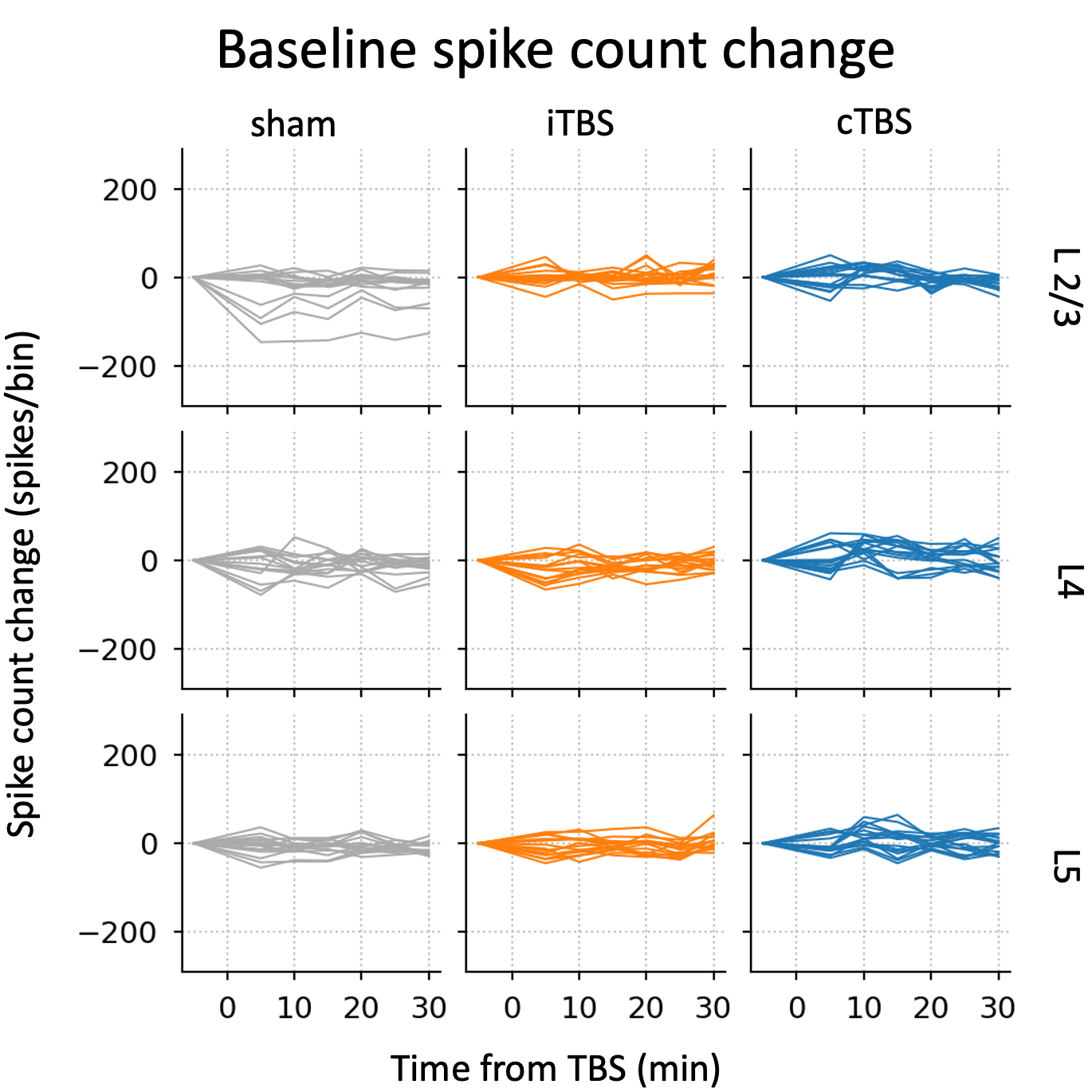

Supplement: Figure 8-1 — Changes in baseline spike count (the number of spikes occurring 5–50 ms before stimulation) after iTBS/cTBS delivery. Each line corresponds to a single recording point (16 recording points in total for each layer, 4 animals × 4 channels). Download Figure 8-1, TIF file. [file eneuro-12-ENEURO.0577-24.2025-s007.tif]

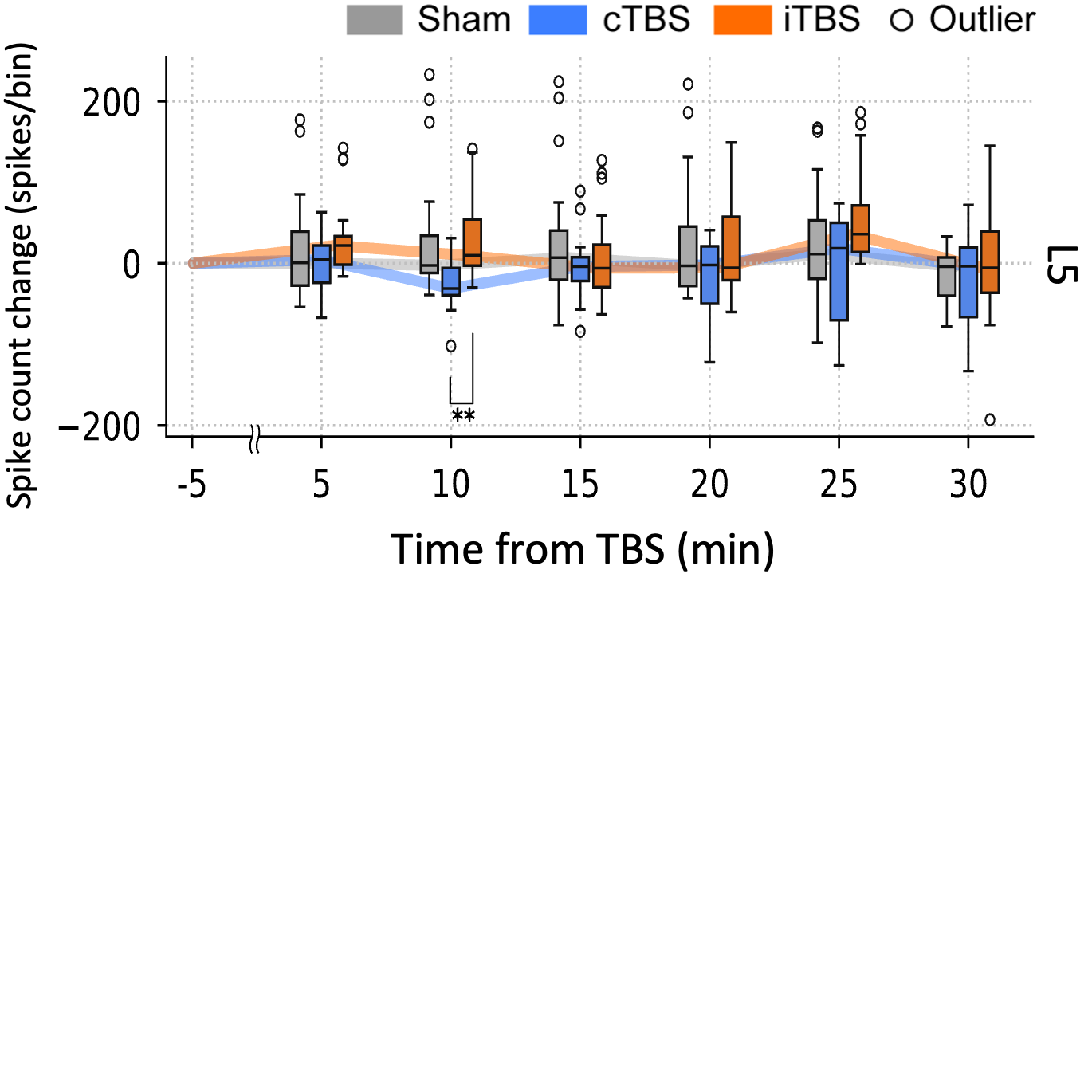

Supplement: Figure 8-2 — Box plots of spike count changes in layer 5. The line plots trace the median spike count changes. Sham (gray), continuous theta-burst stimulation (cTBS; blue), and intermittent theta-burst stimulation (iTBS; orange) groups are shown, with outliers indicated by circles. **p < 0.01 (Kruskal–Wallis test followed by post hoc Steel–Dwass test). Download Figure 8-2, TIF file. [file eneuro-12-ENEURO.0577-24.2025-s006.tif]

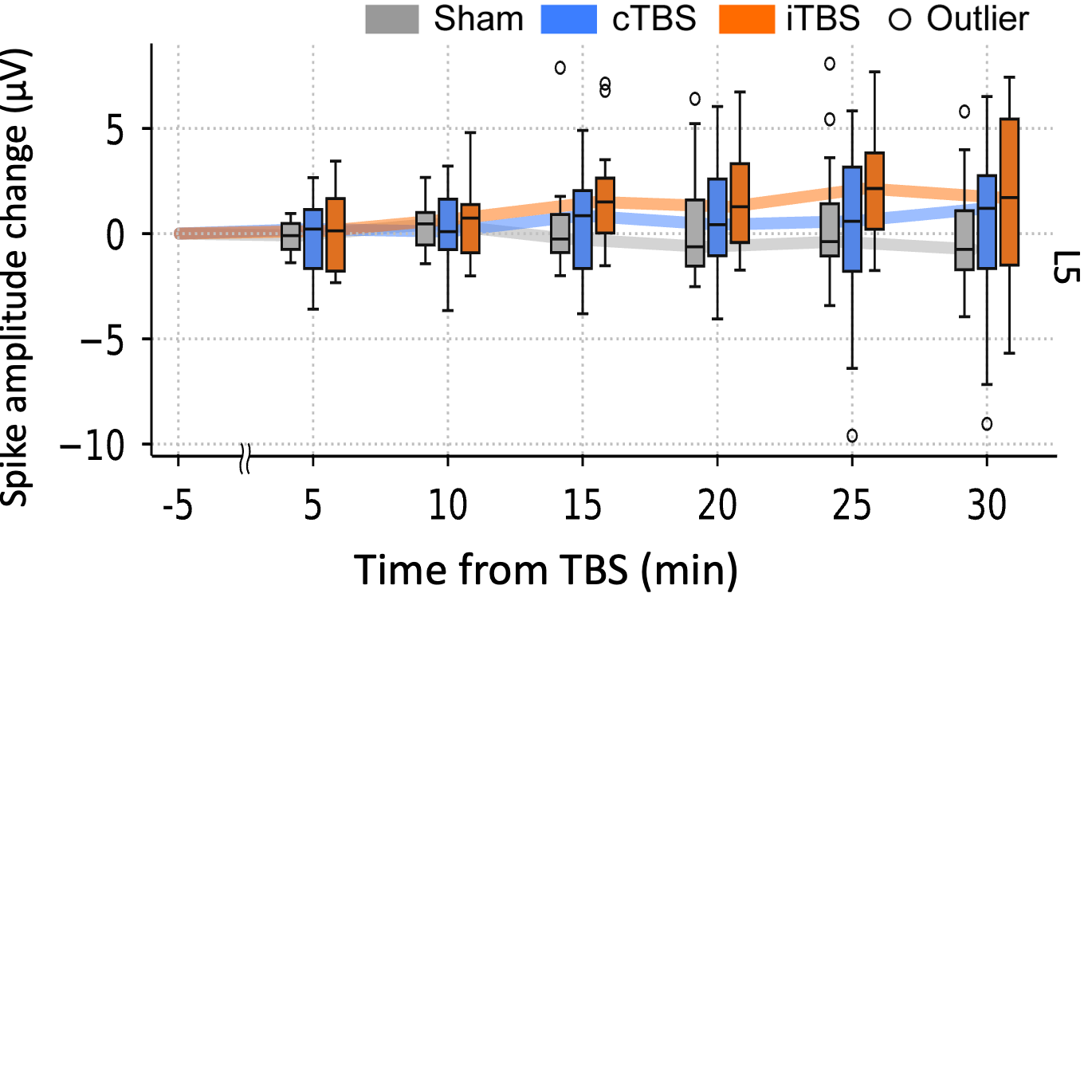

Supplement: Figure 9-1 — Box plots of spike amplitude changes in layer 5. The line plots trace the median spike count changes. Sham (gray), continuous theta-burst stimulation (cTBS; blue), and intermittent theta-burst stimulation (iTBS; orange) groups are shown, with outliers indicated by circles. Download Figure 9-1, TIF file. [file eneuro-12-ENEURO.0577-24.2025-s008.tif]
